# Insight into the biology of *Mycobacterium mucogenicum* and *Mycobacterium neoaurum* clade members

**DOI:** 10.1038/s41598-019-55464-5

**Published:** 2019-12-17

**Authors:** Phani Rama Krishna Behra, B. M. Fredrik Pettersson, Malavika Ramesh, Santanu Dasgupta, Leif A. Kirsebom

**Affiliations:** Department of Cell and Molecular Biology, Box 596, BMC, Uppsala University, SE 751 24 Uppsala, Sweden

**Keywords:** Computational biology and bioinformatics, Evolution, Microbiology, Molecular biology

## Abstract

Nontuberculous mycobacteria, NTM, are of growing concern and among these members of the *Mycobacterium mucogenicum* (*Mmuc*) and *Mycobacterium neoaurum* (*Mneo*) clades can cause infections in humans and they are resistant to first-line anti-tuberculosis drugs. They can be isolated from different ecological niches such as soil, tap water and ground water. Mycobacteria, such as *Mmuc* and *Mneo*, are classified as rapid growing mycobacteria, RGM, while the most familiar, *Mycobacterium tuberculosis*, belongs to the slow growing mycobacteria, SGM. Modern “omics” approaches have provided new insights into our understanding of the biology and evolution of this group of bacteria. Here we present comparative genomics data for seventeen NTM of which sixteen belong to the *Mmuc*- and *Mneo*-clades. Focusing on virulence genes, including genes encoding sigma/anti-sigma factors, serine threonine protein kinases (STPK), type VII (ESX genes) secretion systems and mammalian cell entry (Mce) factors we provide insight into their presence as well as phylogenetic relationship in the case of the sigma/anti-sigma factors and STPKs. Our data further suggest that these NTM lack ESX-5 and Mce2 genes, which are known to affect virulence. In this context, *Mmuc*- and *Mneo*-clade members lack several of the genes in the glycopeptidolipid (GLP) locus, which have roles in colony morphotype appearance and virulence. For the *M. mucogenicum* type strain, *Mmuc*^T^, we provide RNASeq data focusing on mRNA levels for sigma factors, STPK, ESX proteins and Mce proteins. These data are discussed and compared to in particular the SGM and fish pathogen *Mycobacterium marinum*. Finally, we provide insight into as to why members of the *Mmuc*- and *Mneo*-clades show resistance to rifampin and isoniazid, and why *Mmuc*^T^ forms a rough colony morphotype.

## Introduction

Mycobacteria inhabit various environmental reservoirs such as ground and tap water, soil, animals and humans. They are divided into rapid and slow growing mycobacteria, RGM and SGM, respectively. Among SGM, *Mycobacterium tuberculosis* (*Mtb*) causes tuberculosis (TB) whereas the RGM *Mycobacterium smegmatis* MC^2^-155 (*Msmeg*) is frequently used as a mycobacterial model system. As other bacteria, mycobacteria form biofilms, show changes in their colony morphotype (CM) and cell shape. They also appear to have a growth advantage in water that contains disinfecting agents. Some mycobacteria such as *Mycobacterium mucogenicum* (*Mmuc*) is omnipresent in water; it can be isolated from sewage and hospital water systems and has been demonstrated to be associated with various infections^1–6^. The phylogenetically close RGM *Mycobacterium neoaurum* (*Mneo*) and *Mycobacterium cosmeticum* (*Mcos*) were originally isolated from soil and granulomatous lesion of a female patient, respectively^3^.

We recently provided a comparative genomic analysis of non-tuberculosis mycobacteria (NTM) belonging to the *Mmuc*- and *Mneo*-clade emphasizing tRNA and non-coding (nc) RNAs. In addition to *Mmuc*, the *Mmuc*-clade includes *Mycobacterium phocaicum* (*Mpho*), *Mycobacterium aubagnense* (*Maub*) and *Mycobacterium llatzerense* (*Mlla*) while *Mneo* and *Mcos* belong to the *Mneo*-clade^7^. These rapid growing NTM are associated with various infections and they show high tolerance against the first-line anti-tuberculosis drugs isoniazid, rifampin and pyrazinamide^3,5,7–12^ (and Refs therein) and can also show unusual patterns of antibiotic susceptibility^13^. Together this emphasizes the importance of this group of NTMs and provided an incentive for a comparative genomic analysis focusing on virulence and selected regulatory genes. Moreover, information about transcription of genes in NTM, other than *Msmeg* are limited (but see the recent *Mneo* transcriptome and proteome study^14^). Hence, we decided to focus on *Mmuc*^T^, for which the complete genome is available^7^, and study mRNA levels at different *in vitro* growth conditions of selected genes suggested to be involved in gene expression and virulence.

Comparative genomic analysis encompassing seventeen genomes provides new insights into how some of the characteristics of these mycobacteria such as common and unique genes (and their functional classification), and horizontal gene transfer (HGT) might be manifested as phenotypic differences. Specifically, for virulence genes we provide data related to the distribution of sigma factor genes, serine threonine protein kinase (STPK) genes, type VII secretion systems (ESX genes) and mammalian cell entry genes (*mce*). Our data, where we analyzed the mRNA levels for these genes in *Mmuc*^T^ by RNASeq, further suggested that their levels depend on growth conditions and these data are discussed particularly in relation to the SGM *Mycobacterium marinum*, a model system for *Mtb*^15–17^. On the basis of our genomic and RNASeq data we also discuss possible reasons as to why the *Mmuc*- and *Mneo*-clade members show resistance to the first-line anti-tuberculosis drugs rifampin and isoniazid. Finally, we discuss the formation of rough and smooth colony morphotypes among these mycobacteria.

## Results

Members of the *Mmuc*- and *Mneo*-clades share a common ancestor and are phylogenetically close where the *Mmuc*-clade members constitute an earlier branch than the *Mneo*-clade (Fig. S1; Pettersson *et al*. unpublished)^7,11^. We first discuss the overall functional classification of common and unique genes in *Mmuc*- and *Mneo*-members focusing on the type strains *Mmuc*^T^, *Mpho*^T^, and *Maub*^T^ belonging to the *Mmuc*-clade, and *Mneo*^T^ and *Mcos*^T^ members of the *Mneo*-clade (see Methods). We also include *Mycobacterium* sp. URHB0044, which is phylogenetically close to the *Mmuc*- and *Mneo*-clades but does not appear to belong to either of these two clades (Fig. S1). Then, we identify genes acquired through horizontal gene transfer, HGT, and their functional classification. Following this, our analysis focuses on virulence and regulatory genes with a specific emphasis on mammalian cell entry factors (*mce*), type VII secretion (ESX systems) factors, transcription sigma/anti-sigma factors and serine threonine protein kinases (STPK). Finally, we discuss transcription of selected genes in *Mmuc*^T^ (for which the complete genome is available^7^) under different *in vitro* growth phases focusing on virulence genes. In this context, we compare the transcription patterns in *Mmuc*^T^ and in the SGM pathogen *M. marinum* CCUG 20998 (a derivative carrying the gene coding for the red fluorescent protein, *rfp*, and referred to as *Mmar*^rfp^ while *Mmar*^T^ lacks *rfp*)^18^.

### Core and unique gene analysis and functional classification

**Figure 1:**
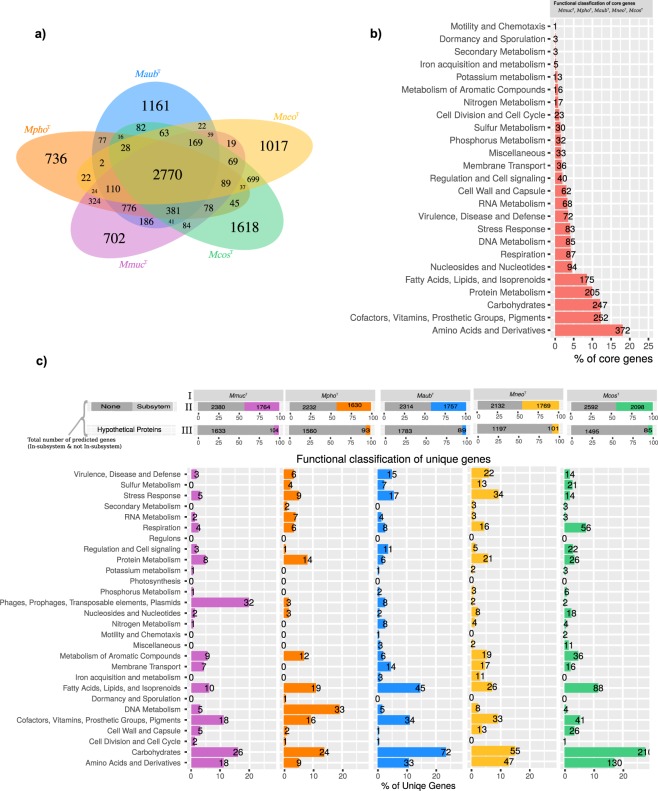
Analysis of CDS and functional classifications. (**a**) Venn diagram showing the presence of common and unique genes for the five type strains *Mmuc*^T^, *Mpho*^T^, *Maub*^T^, *Mneo*^T^, and *Mcos*^T^. (**b**) Functional classification of 2770 core genes present in five type strains *Mmuc*^T^, *Mpho*^T^, *Maub*^T^, *Mneo*^T^, and *Mcos*^T^. (**c**) I, species as indicated. II, grey boxes represent number of genes not functionally classified, and boxes marked with different colors represent number of functionally classified genes in subsystems in the different species as indicated. III, grey boxes represent number of genes classified as hypothetical genes, which are not functionally classified while 104, 93, 89, 101 and 85 correspond to the number of hypothetical genes that are functionally classified. The scale 0 to 100 represent the distribution expressed as percentage, *e.g*. in II (*Mmuc*^T^ column) of the total number of genes (2390 + 1764 = 4154) 42.5% were functionally classified in subsystems. Functional classification of unique genes identified in panel (a) from the five type strains *Mmuc*^T^, *Mpho*^T^, *Maub*^T^, *Mneo*^T^, and *Mcos*^T^.

Homologous and non-homologous chromosomal genes were predicted using the PanOCT (see Methods) and 2770 genes, corresponding to ≈52% of all predicted *Mmuc*^T^ genes, were also identified in *Mpho*^T^, *Maub*^T^, *Mneo*^T^ and *Mcos*^T^ (Fig. 1a). The predicted number of unique genes in *Mmuc*^T^, *Mpho*^T^, *Maub*^T^, *Mneo*^T^ and *Mcos*^T^ were 702, 736, 1161, 1017 and 1618, respectively where *Mcos*^T^ has the highest number of unique genes, which correlates with its larger genome size. The number of genes unique to the *Mmuc*- and *Mneo*-clade members were predicted to be 776 and 699, respectively (Fig. 1a). However, including other *Mmuc*- and *Mneo*-clade members (Fig. S1; Table S1) the numbers dropped to 637 and 557, respectively.

Functional classification of the predicted 2770 core CDSs in the five type strains (*Mmuc*^T^, *Mpho*^T^, *Maub*^T^, *Mneo*^T^ and *Mcos*^T^) did not reveal any major species-specific difference [Fig. 1b; the data set for *Mmuc*^T^ represents all five species and 2054 (of these 1206 were classified in only one category) of the 2770 CDSs could be classified; Fig. S2a]. Subsystem classification suggested that 18% belongs to the “Amino Acids and Derivatives” category, 12% to the “Cofactors vitamins prosthetic groups pigments” category, 12% to the “Carbohydrates” category and 10% to the “Protein Metabolism” category while 2.6% belongs to the “Virulence, Disease and Defense” category. Together, these categories encompass ≈55% of the 2770 core CDSs. Considering unique genes (Fig. 1c) in the same five species revealed that the number of CDSs in the “Carbohydrates” category was higher in *Mcos*^T^ (28%) and in *Maub*^T^ (23%) compared to *Mmuc*^T^ (16%), *Mpho*^T^ (14%) and *Mneo*^T^ (15%). At the “clade” level (Fig. S2b), the Carbohydrates category was 28% (*Mneo*-clade) and 14% (*Mmuc*-clade). Comparing the *Mmuc*- and *Mneo*-clades at the subcategory level, the “Carbohydrates” category “Monosaccharides” was higher in the latter. Also, the data predicted that CDSs in the subcategories “One carbon metabolism”, “CO_2_ fixation and Organic acids” were only detected in the *Mneo*-clade and the number of CDSs in these subcategories was high in both *Mneo*^T^ and *Mcos*^T^ (Fig. S2c). Functional classifications for all 17 *Mmuc*- and *Mneo*-clade members, and *M. sp*. URHB0044, *Mtb*H37Rv, *Msmeg* and *Mmar*^T^, see Fig. S2a.

### Horizontal gene transfer - HGT

**Figure 2:**
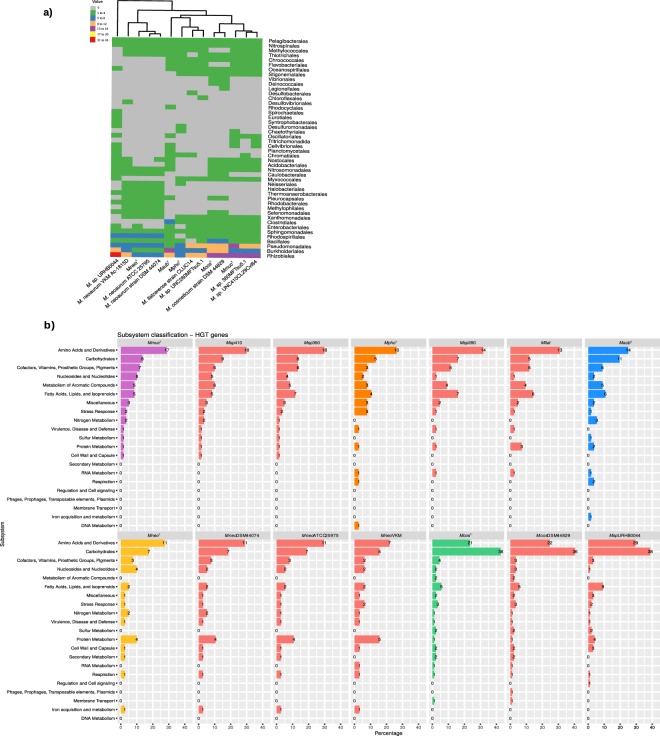
Identification of horizontally transferred genes and their functional classification. (**a**) Heat map plot showing putative horizontally transferred genes in the *Mmuc*- and *Mneo*-clade members (species indicated below the heat map plot) using HGTector (see Methods). The upper left shows the colored code where the x-axis represents the number of horizontally transferred genes; the scale is in multiple of 4 (green, 1 to 4; blue, 5 to 8; orange, 9 to 12; purple, 13 to 16; yellow, 17 to 20; red, 21 to 24) genes originating from different orders indicated to the right, *e.g*. *Pelagibacterales*. Grey refers to that no genes originate from indicated order. (**b**) Functional classification of putative horizontally transferred genes in the *Mmuc*- and *Mneo*-clade members.

To predict horizontally transferred genes we used HGTector (see Methods) and the number of genes ranged from 41 (*Mneo* ATCC25975 referred to as *Mneo*^ATCC25975^) to 88 (*M. sp*. URHB0044), see Table S1. For the five type strains, *Mmuc*^T^, *Mpho*^T^, *Maub*^T^, *Mneo*^T^ and *Mcos*^T^, we predicted that of the six HGT genes common to these mycobacteria, five genes are derived from Proteobacteria (two from the β-, two from the γ-, one from the α-branch) and one from unclassified bacteria. Many HGT genes in the five type strains were predicted to originate from several other bacterial sources with the highest numbers from *Rhizobiales, Burkholderiales* and *Pseudomonadales* (Fig. 2; Table S2a–p). In *Mmuc*^T^ and *Mpho*^T^ we also detected genes of possible eukaryotic origin; the inosine-5-monophosphate dehydrogenase gene (MMUC_02941, which probably originates from *Tritrichomonas suis*), pseudooxynicotine oxidase (MMUC_05484 and MPHO_05174) gene, FAD dependent oxidoreductase (MPHO_04106) gene and a gene encoding a hypothetical protein in *Mneo*^T^ (MNEO_01334). The eukaryotic origin for the three latter genes was not identified (Table S2a–p). Functional classification of HGT genes in 14 of the mycobacteria including the type strains, revealed that the highest number of genes were to be found in the category “Amino Acids and Derivatives” with the notable exception of *Mcos*^T^ and *M. sp*. URBH0044. For these two, roughly 40% of the HGT genes belong to the category “Carbohydrates” (Fig. 2b).

### Virulence genes

**Figure 3:**
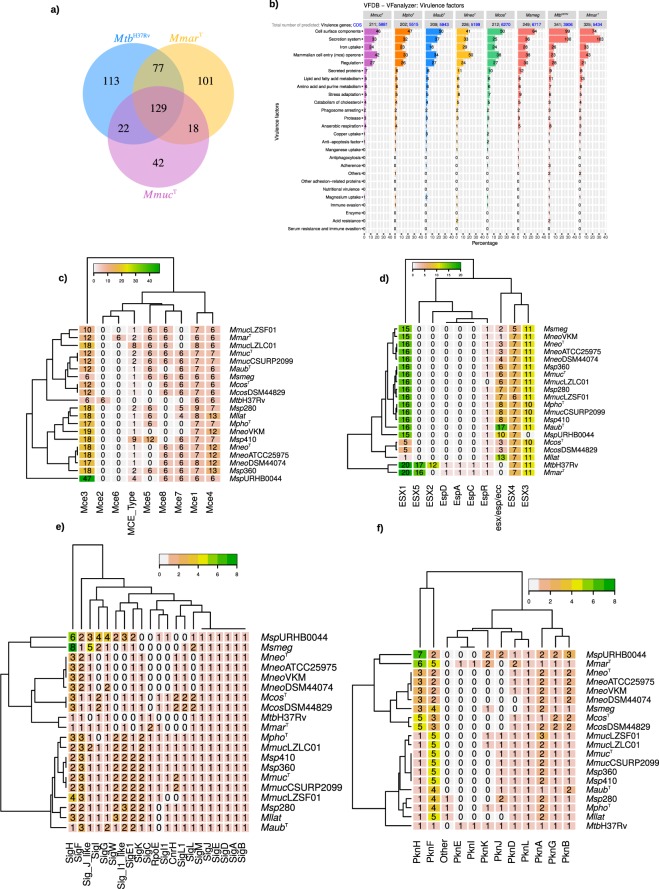
Virulence factors and analysis of selected genes related to virulence. (**a**) Venn diagram showing common and unique homologous virulence factor genes among *Mmuc*^T^, *Mtb*H37Rv, and *Mmar*^T^. (**b**) Classification of virulence factors genes (VFanalyzer, VFDB; see Methods) present in *Mmuc*^T^, *Mpho*^T^, *Maub*^T^, *Mneo*^T^, *Mcos*^T^, *Mtb*H37Rv, *Mmar*^T^, and *Msmeg* as indicated. (**c**) Heat map plot showing the number of genes corresponding to the different *mce* operons, marked as Mce1 to Mce8, among *Mmuc*- and *Mneo*-clade members, *Mtb*H37Rv, *Mmar*^T^ and *Msmeg*. MCE_type represents *mce* related genes that could not be classified as Mce1-8. (**d**) Heat map plot showing the distribution of different ESX genes among *Mmuc*- and *Mneo*-clade members, *Mtb*H37Rv, *Mmar*^T^ and *Msmeg*. (**e**) Heat map plot showing the distribution of sigma factor genes among *Mmuc*- and *Mneo*-clade members, *Mtb*H37Rv, *Mmar*^T^ and *Msmeg*. (**f**) Heat map plot showing the distribution of STPK genes among *Mmuc*- and *Mneo*-clade members, *Mtb*H37Rv, *Mmar*^T^ and *Msmeg*.

The *Mmuc*- and *Mneo*-clade members carry virulence genes (Table S1) and 129 genes are common for *Mmuc*^T^, *Mtb*H37Rv and *Mmar*^T^ (Fig. 3a). Compared to the non-pathogen *Msmeg*, roughly similar numbers of genes in *Mmuc*- and *Mneo*-clade members were predicted while *Mtb*H37Rv and *Mmar*^T^ encode higher numbers. Among these, genes in the categories: cell surface components, secretion systems and *mce* operons are abundant (Figs. 3b and S3; Table S3a). Specifically, we noted that the numbers of predicted *mce* homologs are slightly higher in *Mmuc*- and *Mneo*-clade members compared to *Mtb*H37Rv while *Mtb*H37Rv and *Mmar*^T^ have higher numbers in the categories “Cell Surface Components” and “Secretion Systems”. We also noted that *Mmuc*- and *Mneo*-clade members lack *mce*2 homologs (see above; Fig. 3c; Table S3b), which influence *Mtb* virulence^19^. In the category secretion systems, homologs of ESX-5 associated genes (Fig. 3d; Table S3c), known to have an impact on mycobacterial virulence^20,21^, are missing in the *Mmuc*- and *Mneo*-clade members (see below). With respect to the category regulation, we predicted the number of transcription sigma (σ) factor genes and serine-threonine protein kinase (STPK) genes, which have key roles in global regulation of gene expression and function of proteins and hence virulence^22,23^. Briefly, the numbers of σ-factor genes vary between 17 and 29 among members of the *Mmuc*- and *Mneo*-clade (*M. sp*. URHB0044 has 35; see below). This should be compared to *Mtb*H37Rv and *Mmar*, which carries 13 and 17–18 (dependent on strain) σ-factor genes, respectively^18,22^. Interestingly *sigC*, mainly present in SGM such as *Mtb*H37Rv and *Mmar*, is present in the *Mmuc*-clade members but absent in the *Mneo*-clade (Fig. 3e; Table S3d). In this context, we note that *Msmeg* encode 28 σ-factors (for further details, see below).

Another group of regulatory genes, STPK genes, was also predicted to be larger compared to *Mtb*H37Rv (Fig. 3f; Table S3e; see below), which might be related to that *Mmuc*- and *Mneo*-clade members are found in different ecological niches than that of *Mtb*. The higher numbers were mostly attributed to multiple copies of *pknF* and/or *pknH*.

Given that *Mmuc*^T^ shows a mucoid growth we searched for the presence of genes reported to influence formation of rough and smooth colony morphotype (CM) focusing on genes constituting the GPL locus and involved in the synthesis of glycopeptidolipids that are present in the cell wall and as such have a role in virulence^24^. Interestingly, members of both the *Mmuc*- and *Mneo*-clades lack several GPL locus genes suggesting differences in the nature of the glycopeptidolipids in these mycobacteria compared to *Mtb*, *Mycobacterium abscessus* and *Msmeg* (Table S3f; see discussion). In this context, we note that all *Mmuc*-clade members lack *ahpC*, suggested to influence resistance against the antibiotic isoniazid^25^, while it is present in the *Mneo*-clade members. Also, *sitA* and *sitB* (genes related to iron uptake in *e.g*. *Shigella flexneri*^26^), and *ureB* and *ureG* (genes related to acid resistance and nitrogen metabolism^27^) were predicted to be present in *Mneo*-clade members but not in the species of the *Mmuc*-clade (Table S3a). For members of the *Mmuc*-clade we also detected the presence of nitrate reductase related genes (anaerobic respiration). Finally, *mosR*, a redox-dependent transcriptional repressor identified in *Mtb* reported to influence virulence^28^, appears not to be present in any of these NTM (Table S3a).

### Mammalian cell entry – mce – operons

*Mtb*H37Rv has four *mce* operons, *mce*1-4, while ten *mce* gene clusters have been predicted in the environmental mycobacteria *Mycobacterium phlei*^19,29^. The *Mmuc*- and *Mneo*-clade members also carry *mce* genes (Fig. 3c; Table S3b). Briefly, *mce*1, 3 and 4 are present in all the genomes, while *mce*7 (and *mce*8) is missing in *Mpho*^T^ and *Mneo*^VKMAc-1815D^. The *mce*5 operon appears to be missing in the *Mneo*-clade members, while it is present in members of the *Mmuc*-clade. As discussed above, none of the studied mycobacteria was predicted to carry the *mce*2 operon but some were predicted to have a few *mce*2 paralogs, *e.g*., two *mce*2 paralogs were predicted in *Mmuc*^T^ (Table S3b). Also, we noticed the presence of two or three *mce*3 operons in several of the genomes as well as an extra *mce*4 operon in several of these mycobacteria (Fig. 3c; Table S3b). Taken together, as in *Mtb*H37Rv, these NTM carry *mce* operons that relate to virulence, cell wall lipid composition (*mce*1), and lipid metabolism (where *mce*3 relates to lipid metabolism and *mce*4 to cholesterol metabolism^29–34^).

### Type VII secretion – ESX-operons

We predicted the presence of ESX gene families from the different mycobacterial species using the BLASTp approach and *esx* genes present in the *Mtb*H37Rv genome (see Methods). For comparison, we included *Mmar*^T^ and *Msmeg*. Following this outline, we predicted homologs to the ESX-1, ESX-3 and ESX-4 gene clusters for members of the *Mmuc*- and *Mneo*-clades, and *M. sp*. URBH0044, while ESX-2 and ESX-5 genes seem to be absent in these NTM (Fig. 3d; Table S3c). Moreover, we could not predict *Mtb*H37Rv orthologs of the *espACD* operon, known to have a role in virulence^20,21^, in any of the *Mmuc*- and *Mneo*-clade members (or *Msmeg*) genomes. But, paralogs to these genes, except *espC*, were predicted in some of the genomes, *e.g*. *Mpho*^T^ (Fig. 3d; Table S3c). These might be homologs of *espE*, *espF* and *espG* but using a bidirectional best hit analysis approach, with *Mtb*H37Rv as reference, this appears not to be the case (not shown). Moreover, several of the genes in the ESX-1 loci in *Mcos*^T^ appear to be absent while compared to *Mtb*H37Rv, the ESX-1 genes *espE*, *espF*, *espJ* and *espK* are missing in all the *Mmuc*- and *Mneo*-clade members (Table S3c). Interestingly, *pknJ* (encoding a STPK, see above) is located in the ESX-1 region in members of the *Mmuc*-clade, however, its role with respect to secretion (if any) is not known.

### Sigma and anti-sigma factor genes

For *Mmuc*^T^, *Mpho*^T^, *Maub*^T^, *Mneo*^T^ and *Mcos*^T^ the predicted number of σ-factor genes ranged between 17 and 29, with *Mmuc*^T^ and *Mneo*^T^ having the highest and lowest numbers, respectively (Fig. 3e; Table S3d). The numbers for the other *Mmuc*- and *Mneo*-clade members were similar while for *M. sp*. URBH0044 we predicted 35 σ-factor genes. So far, this is the highest number of σ-factor genes predicted in any mycobacteria. Compared to the other mycobacteria, its genome is larger (≈7.5 Mbp) and it does not belong to either the *Mmuc*- or the *Mneo*-clade^7^.

Sigma factors are divided into four groups; Group 1 and 2 include the housekeeping σ-factor, SigA, and SigB, respectively, group 3 SigF and group 4 the ECF (extra cytoplasmic function) σ-factors^35–38^. As other mycobacteria *Mmuc*- and *Mneo*-clade members code for several ECF σ-factors in addition to SigA, SigB and SigF. Single copies were detected for *sigA*, *sigB*, *sigC* (when present), *sigD* and *sigM* irrespective of species (Fig. 3e; Table S3d). The majority of the σ-factor genes belong to group 4, the ECF^22,37,38^. For several of these, more than one gene was annotated, as exemplified by σ-factor E (*sigE*) with three copies (four including *rpoE*), and with two copies of *sigH*, *sigK* and *sigL* in *Mmuc*^T^. *Mmuc*-clade members were also predicted to have three copies of the alternative σ-factor *sigF*. In Table S3d, the σ-factors with more than one copy annotated are listed separately based on gene synteny (*e.g*., *sigF* genes were predicted at three different genomic locations; Fig. S4a,b). Moreover, in keeping with that *Mneo*-clade members have fewer σ-factor genes the numbers of extra copies of different alternative σ-factor genes are lower. As discussed above, *sigC* was predicted in members of the *Mmuc*-clade, including the type strain *Mmuc*^T^, while it is missing in members of the *Mneo*-clade (Fig. 3e; Table S3d; see discussion). This sigma factor has previously been reported to exist in slow growing pathogenic mycobacteria such as *Mtb*H37Rv and *Mmar* but not in the RGM *Msmeg*^22,37,38^ (and Refs therein). Also, *sigC* is implicated to have a role in *Mtb* pathogenicity^39–42^. Whether this is the case also for *Mmuc*-clade members warrants further studies.

Gene synteny analysis suggested that *sigD*, *sigE*1, *sigF*3, *sigH*2, *sigK*1, *sigK*2, *sigL*1 and *sigM* are closely linked to genes encoding the corresponding putative anti-σ factor (see below). Thus, we consider these σ-factor genes as homologs (except for the two *sigK* genes both of which are positioned close to their respective anti-σ factor genes) of the single σ-factor genes in other mycobacteria such as *Mtb*H37Rv and *Mmar*^18,37,38^. Alignment of the different σ-factors indicated variations within the respective group (Fig. S4c). To understand the interrelation between the σ-factors we therefore generated a “Sigma factor” phylogenetic tree (based on amino acid sequence). In this tree, the σ-factors are deployed in distinct clusters (Fig. S4d). The SigF variants cluster (close to SigA and SigB) indicating that these indeed belong to group 3 and have a common ancestor. For the ECF group and using the *Mtb* ECF σ-factors as reference the following are suggested to be phylogenetically close: i) SigH and SigE, ii) SigM and SigC1, iii) SigK (K1 and K2) and SigL, iv) SigI1 and SigI1-like, v) SigL1, SigE1 and SigW, vi) CnrH, RpoE, and SigG, and vii) SigJ, SigI and SigJ-like while SigD forms a separate cluster. Of notice, the SigI1 and SigI1-like cluster at a different location (close to SigL and SigL1) than SigI. Together these findings indicate the phylogenetic relationship and evolutionary history of the σ-factors in these mycobacteria.

The activities for several of the ECF σ-factors such as SigC, SigD, SigE, SigF, SigH, SigK, SigL and SigM are regulated by anti-σ factors (Fig. S4e). The anti-σ factor genes co-localize with the σ-factor genes with the exception of the putative *rscA* (anti-SigC)^37,38,43^, which is positioned elsewhere on the chromosome in *e.g*, *Mtb*^44^. However, in *Mmuc*-clade members it is located close to *sigC* with four or five genes in between (Fig. S4f; see discussion). Moreover, as in *Mtb* the anti-σ factor genes *rsbW*, *rsdA*, *rseA*, *rskA* (*A*1 and *A*2), *rsmA*, *rslA*, *rshA* are directly linked to the corresponding σ-factor gene in the *Mmuc*- and *Mneo*-clade members (Table S3g). An “anti-Sigma factor” phylogenetic tree (based on amino acid sequences with RsbW as the root) revealed that RsdA is closest to the ancestor followed by RskA1 and RskA2, RsmA, RslA, RshA and RseA. This is in rough agreement with the phylogeny for the corresponding σ-factors with the notable exceptions for RsdA and RseA. The RsdA is closer to the two RskA, RscA, RslA, RsmA and RshA while SigD (using SigF as the root) branch-out earlier and is positioned closer to the rooted σ-factor. The opposite is the case for RseA, which is positioned closer to the rooted anti-σ factor whereas SigE shares a common ancestor with SigH and is positioned closer to SigC, SigM, SigL and the two SigK (Fig. S4g,h; see discussion).

### Serine threonine protein kinases – STPKs

We predicted a total of 35 different *pkn* genes encoding STPKs in all the members of the *Mmuc*- and *Mneo*-clades (Fig. 3f; Table S3e). Following the naming of STPK genes identified in *Mtb*H37Rv^23^, these *pkn* genes were predicted to be orthologous of *pknA-L*, or to one referred to as *pkn*. The number of *pkn* genes in the individual species varied from 14 (*Mneo*^T^; notably, only 13 genes were predicted using the *Mneo*^DSM44074^ genome available at NCBI) to 19 genes (*Mmuc*^LZSF01^; which is a *Mpho* strain^7^), while *M. sp*. URBH0044 with its larger genome was predicted to carry 24 *pkn* genes. This should be compared to *Mtb*H37Rv, *Mmar*^T^ and *Msmeg*, which harbor 11, 27 (21 with and six genes lacking the kinase domain) and 16 *pkn* genes, respectively (Fig. 3f; Table S3e). Genes encoding PknB, PknG and PknL were detected in all species, including *Mtb*H37Rv, *Mmar*^T^ and *Msmeg*, indicating their importance in mycobacteria. PknA is another important STPK^23^, and we predicted *pknA* in all these mycobacteria, except *Mcos*^T^. However, we detected it in the publicly available *Mcos*^DSM44829^ genome. Therefore, its absence in our *Mcos*^T^ genome is likely due to draft genome status. Interestingly, we predicted two copies of *pknA* and *pknB* in the *Mneo*-clade members. Two *pknA* genes were also found in *Mmuc*^T^ and *Mpho*^T^, while one *pknA* and two *pknB* were detected in *Maub*^T^ (failure to detect the additional *pknA* copy in *Maub*^T^ could again be due to draft genome status). Of the two *Mmuc*^T^ *pknA* (*pknA*1 and *pknA*2; 37% identity and 86% query coverage), *pknA*1 is positioned downstream of *pknB* while *pknA*2 is localized elsewhere on the chromosome. An extra *pknG* gene was also predicted in *Mcos*^T^, while *pknK* was only detected in *Mpho*^T^ and *Mmuc*^LZSF01^. Multiple copies of *pknF* were predicted to be common in the *Mmuc*-clade members, while several *pknH* copies were found in *Mneo*-clade members. On the basis of these data (Supplementary information Table S3e, *pkn* genes as indicated in columns C and F) we generated the “Pkn phylogenetic tree” illustrating the interrelation between the different STPKs in these mycobacteria and *Mtb*H37Rv (Fig. S4i; see also ref. ^45^).

### Analysis of transcription of selected virulence genes in *Mmuc*^T^ under different growth conditions

**Figure 4:**
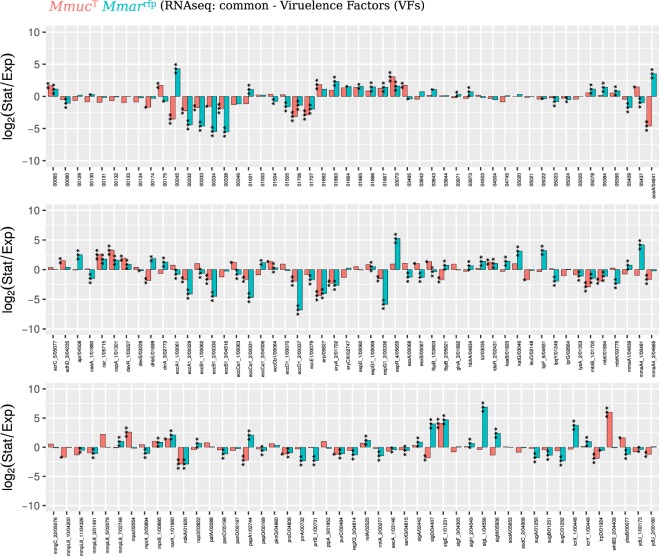
Transcription of virulence genes in *Mmuc*^T^ and *Mmar*^rfp^ Bar plot showing mRNA levels for 147 virulence factor genes present in exponentially growing and stationary *Mmuc*^T^ (red) and *Mmar*^rfp^ (turquoise) cells as indicated. A negative log_2_-value suggests that the corresponding mRNA is more abundant in exponentially growing cells while a log_2_-positive value suggest higher levels in stationary cells. The x-axis labels refer to the gene name and/or gene annotation number in *Mmuc*^T^. Statistical significance, see Methods; *p < 0.05; **p < 0.01; ***p < 0.001. For the genes where no genes are indicated (top panel) the numbers refer to annotated gene numbers in *Mmuc*^T^ since no gene names are available. For a detailed description see Table S3a.

To map transcription, we isolated RNA from *Mmuc*^T^ cells growing at exponential phase and cells from stationary phase and subjected the RNA to RNASeq (see Methods). For comparison, we decided to use the SGM *Mmar*^rfp^ for which we have access to similar transcriptome data^18^ (unpublished data). We focused on 129 virulence genes identified above (Fig. 3a,b) and the data are presented in Fig. 4.

For the majority of genes, the change (log_2_-fold) in mRNA levels comparing exponentially growing cells with stationary cells showed similar patterns (albeit the magnitudes of change differ) for *Mmuc*^T^ and *Mmar*^rfp^ with a few notable exceptions. In *Mmuc*^T^, the *mmgC*_4 transcript is higher in stationary phase while in *Mmar*^rfp^ it is lower. This is also the case for *mmaA*4_1, *aceA*, *papA*1, *fbpB*_2, and *dhbE* mRNA. All these genes are related to building the cell wall. Considering σ-factor mRNAs (with roles in virulence, see also below), we noted that the levels for *sigD*, *sigL*_1 and *sigM* in *Mmuc*^T^ are higher in stationary cells while in *Mmar*^rfp^ the corresponding transcripts are either unchanged (*sigL*_1) or higher (*sigD* and *sigM*) in exponentially growing cells (Fig. 4; see also below)^18^. With respect to antibiotic resistance, transcripts of *Mmuc*^T^ genes such as *katG* and *lipF* are higher in stationary phase and their levels is also higher compared to *Mmar*^rfp^. Moreover, mRNA levels for the well-studied *esxA* and *esxB* genes are higher in exponentially growing *Mmuc*^T^ cells while for *Mmar*^rfp^ the corresponding transcripts are more abundant in stationary phase (see below). Taken together, it appears that some genes related to virulence are differentially expressed comparing the RGM *Mmuc*^T^ and SGM *Mmar*^rfp^ suggesting variation in the regulatory circuits controlling the expression of these genes and possibly genes under the control of SigD, SigL_1 and SigM.

Below we focus on sigma factor, ESX, *mce* and STPK mRNA levels in *Mmuc*^T^ and compare with the levels detected in *Mmar*^rfp^. We calculated mRNA levels in two ways; distribution refers to the abundance of mRNA for each individual gene relative to the sum of the other transcripts of related genes, *e.g*., level of SigA mRNA relative to the sum of all σ-factor mRNA levels. While change refers to the change (log_2_-fold) in mRNA levels comparing exponentially growing cells and cells in stationary phase.

### Variation in sigma factor transcripts

**Figure 5:**
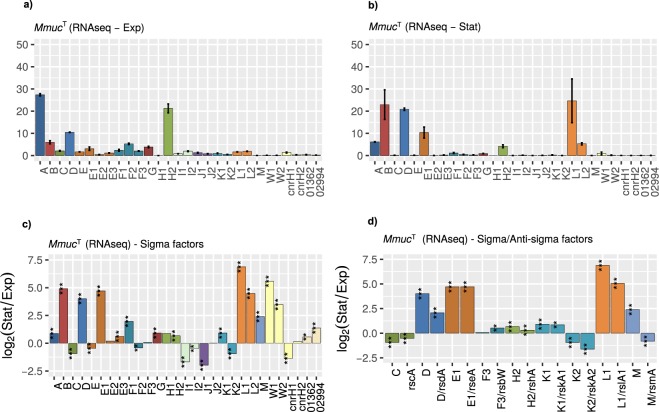
Analysis of sigma factor mRNA levels in exponentially growing and stationary *Mmuc*^T^ cells. (**a**,**b**) Distribution profiles for *Mmuc*^T^ sigma factor mRNAs at different growth conditions expressed as percentage, panel (**a**) exponentially growing cells and panel (**b**) stationary cells. All sigma factor mRNAs together constitute 100%, for details see main text. (**c**) Change, expressed as log_2_-fold change, comparing mRNA levels in exponentially growing and stationary *Mmuc*^T^ cells. A negative log_2_-value suggests that the corresponding mRNA is more abundant in exponentially growing cells while a positive value suggest higher levels in stationary cells. (**d**) Change in mRNA levels (log_2_-fold) for cognate sigma/anti-sigma pairs mRNA levels in exponentially growing and stationary *Mmuc*^T^ cells, see also figure legend (**c**). Statistical significance, see Methods; *p < 0.05; **p < 0.01; ***p < 0.001.

In exponential growth phase, SigA, SigD and SigH2 mRNAs are the most abundant and constitute roughly 60% of the *Mmuc*^T^ σ-factor transcripts while the remaining transcripts are distributed among the other σ-factors (Fig. 5a). Compared to stationary phase there is a notable change such that the levels for SigB, SigE1 and SigL1 mRNAs increased while the fraction of SigA mRNA was lower (Fig. 5b). However, mRNA levels for the majority of σ-factors were higher in stationary cells relative to exponential cells with notable exceptions for *sigC*, *sigE*, *sigF*2, *sigI*1, *sigI*2, *sigJ*1, *sigK*2 and *cnrH*1 (Fig. 5c). Higher levels of SigB and SigE mRNAs in stationary phase are consistent with what we previously reported^18^ for the SGM *Mmar*^rfp^. Moreover, in contrast to the RGM *Mmuc*^T^ the levels for the majority of the σ-factors were found to be higher in exponentially growing *Mmar*^rfp^ cells (Fig. S5a–c)^18^. From this comparison, it appears that the regulation of the expression of the σ-factor genes differs comparing *Mmuc*^T^ and *Mmar*^rfp^. Also, the data indicates that SigB and SigE (SigE1 in *Mmuc*^T^) represent σ-factors necessary for the expression of genes in stationary phase both in RGM and SGM.

Considering anti-σ factor mRNAs, we observed that the change in mRNA levels for *rseA*, *rsdA*, *rshA*, *rskA*1, *rskA*2 and *rslA*1 followed the change for the corresponding σ-factor mRNAs (Fig. 5d). For *rsbW* it was higher, which might be related to that *rsbW* is positioned upstream of *sigF*3 while the other anti-σ factor genes are located downstream of the corresponding σ-factor gene (but note *rscA*). The relationship comparing mRNAs for these sigma factors and anti-sigma factors was expected given that they likely belong to the same transcriptional unit. In contrast to the other “σ/anti-σ“ pairs, the *rsmA* mRNA level is higher in exponentially growing cells while for *sigM* it is higher in stationary phase (Fig. 5d). This might indicate differences in the regulation of *sigM vs rsmA* and/or stability of the corresponding mRNA. We also note that the mRNA level for the putative anti-SigC follow the same trend as SigC (Fig. 5d).

### Variation in the levels of ESX, STPK and Mce mRNAs under different growth conditions

**Figure 6:**
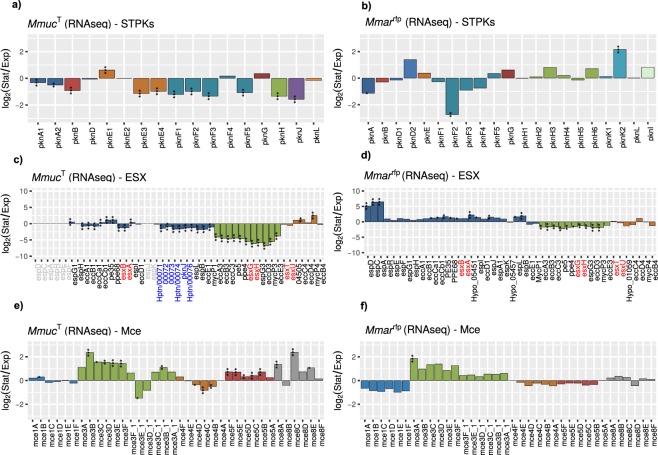
Analysis of STPK, ESX and Mce mRNA levels in exponentially growing and stationary *Mmuc*^T^ and *Mmar*^rfp^ cells. (**a**,**b**) Change, expressed as log_2_-fold change, comparing STPK mRNA levels in exponentially growing and stationary *Mmuc*^T^ (panel a) and *Mmar*^rfp^ (panel b) cells. A negative log_2_-value suggests that the corresponding mRNA is more abundant in exponentially growing cells while a positive value suggest higher levels in stationary cells. (**c**,**d**) Change, expressed as log_2_-fold change (see a and b above), comparing ESX mRNA levels in exponentially growing and stationary *Mmuc*^T^ (panel c) and *Mmar*^rfp^ (panel d) cells. (**e,f**) Change, expressed as log_2_-fold change (see **a**,**b** above), comparing Mce mRNA levels in exponentially growing and stationary *Mmuc*^T^ (panel e) and *Mmar*^rfp^ (panel f) cells. Statistical significance, see Methods; *p < 0.05; **p < 0.01; ***p < 0.001.

Irrespective of growth phase mRNAs originating from *pknA*1, *pknB* and *pknF*2 were the most abundant; together they constitute roughly 50% of all STPK mRNAs. However, comparing mRNA levels in exponential and stationary phase suggested that these as well as the other *pkn* mRNAs decreased in stationary phase with the exception of *pknE*1 mRNA, which increased, but the magnitude of change was less than one-fold (log_2_) (Fig. 6a; for distribution see Fig. S6a,b). Moreover, given that *pknA*1 is positioned upstream of *pknB* and that the *pknB* mRNA is approx. two-fold more abundant (irrespective of growth phase; Fig. 6a) than *pknA*1 might indicate regulation at the transcriptional level or that the *pknA*1 mRNA is more prone to degradation. Compared to *Mmuc*^T^, *Mmar*^T^ carries 21 complete STPK genes (of 27, see above) and *pknA* and *pknB* mRNAs are also among the most abundant in exponential and stationary cells with *pknB* mRNA modestly higher (Fig. S6c,d). As for *Mmuc*^T^, we detected small changes with the notable exceptions for *pknF*2 and *pknK*2, which are higher during exponential growth and in stationary cells, respectively (Fig. 6b). Together this indicated similarities in STPK mRNA levels (*i.e*. the homologs) in these two phylogenetically distant mycobacteria.

Among ESX clusters, transcripts originating from ESX-1 were the most abundant followed by ESX-3 transcripts in *Mmuc*^T^ (Fig. 6c). The ESX-1 transcripts from *esxA* and *esxB* constituted roughly 35% of all ESX-1 transcripts. Moreover, some of the ESX-1 mRNA levels are higher in exponentially growing cells, including *esxB* and *esxA*, while the levels for others such as *pe35* and *ppe68* increase in stationary phase (Fig. 6c). By contrast, the ESX-3 mRNA levels are higher [between approx. 3.8- to 6.8-fold (log_2_)] in cells growing in exponential phase. The levels for ESX-4 transcripts are low but it appears that there is an increase for two ESX-4 genes, *eccC*4 and *mycP*4, in stationary phase. With respect to ESX-1, EccC and MycP (a mycosin protease essential for secretion) are integrated into the inner membrane^46^. For *Mmar*^rfp^, mRNA levels for the ESX-1 genes (except *eccE*1 and *mycP*1) and *espACD* were all higher in stationary phase while changes in the patterns for ESX-3 and ESX-4 mRNAs were similar to those observed in *Mmuc*^T^ (cf. Fig. 6c,d; see discussion).

The distribution profiles in *Mmuc*^T^ for *mce* operon transcripts showed high similarity comparing exponential and stationary cells with the most abundant transcripts originating from the *mce*1 operon followed by the *mce*4 and *mce*5 while the levels for *mce*3, *mce*7 and *mce*8 were low (Fig. S6g,h). Moreover, comparing mRNA levels isolated from exponential and stationary cells revealed an increase for *mce*3 (except for *mce*3E_1 and *mce*3D_1), *mce*5 and *mce*8. The increase was however ≤2.4-fold (log_2_; Fig. 6e). For *Mmuc*^T^, *mce*4 and *mce*7 transcripts we detected lower levels for some of these mRNAs in stationary cells and no change for *mce*1 transcripts (Fig. S6i). The pattern of change for the corresponding *mce* genes in *Mmar*^rfp^ was similar compared to *Mmuc*^T^ (except *mce*8 genes; cf. Fig. 6e,f). On the basis of these observations it appears that these *mce* operons in these two mycobacteria, in particular in *Mmuc*^T^, are differentially expressed dependent on growth phase.

## Discussion

We recently reported that the size of the genomes for *Mmuc*- and *Mneo*-clade members including type strains range between 5.4 to 6.4 Mbp and that phages, IS elements as well as horizontally transferred tRNA genes and phage-derived ncRNAs have likely contributed to the evolution of these mycobacteria^7^. Here we provide data suggesting that the number of unique genes range between 702 and 1618 in the type strains *Mmuc*^T^, *Mpho*^T^, *Maub*^T^, *Mneo*^T^ and *Mcos*^T^ encompassing roughly 12% to 26% (dependent on mycobacteria) of the total annotated CDSs. While comparing *Mmuc*- and *Mneo*-clade members revealed that the former group carry 637 and the latter 557 unique genes (based on sixteen genomes; Fig. S1). Among the unique genes we noted differences such as a notable number of genes classified as: “Phages, Prophages, Transposable elements, Plasmids” in *Mmuc*^T^, “DNA metabolism” in *Mpho*^T^ and “Carbohydrates” and “Respiration” in *Mcos*^T^. Also, *Mneo*-clade members carry a larger number of core genes in the category “Carbohydrates” where *Mcos*^T^ encodes the highest number of unique genes in this category (as well as in the “Amino Acids and Derivatives” category). Together this expands our previous study^7^ and provides further insight into the evolution of these mycobacterial species. As such our findings reflect their capacity to thrive in different environments. In this context, *Mmuc*- and *Mneo*-clade members were predicted to carry several horizontally transferred genes (HGT) originating from other bacteria as well as genes of eukaryotic origin such as the inosine-5’-monophosphate dehydrogenase gene from *Tritrichomonas suis*, a parasite of pigs, cattle and cats^47^.

### Type VII secretion systems

Of the CDSs in the type strains *Mmuc*^T^, *Mpho*^T^, *Maub*^T^, *Mneo*^T^ and *Mcos*^T^ we predicted that approx. 3.4 to 4.4% code for proteins related to virulence. This is in accordance with *Msmeg* while of the 3906 CDSs in *Mtb*H73Rv 8.7% (6% in *Mmar*^T^) are classified as virulence genes. The difference is mainly related to that *Mtb*H73Rv and *Mmar*^T^ both have larger numbers of genes in the categories “Cell surface components” and “Secretion system” while the number of *mce* genes is lower. Of the five type VII secretion systems (ESX 1-5) identified in *Mtb*H37Rv and *Mmar* (which also has ESX-6 but no ESX-2), ESX-1, -3 and -5 have been reported to affect virulence^20,46,48–52^. The *Mmuc*- and *Mneo*-clade members lack ESX-5 genes but carry ESX-1 and ESX-3. With respect to ESX-1 *espE*, *espF*, *espJ* and *espL* are missing where *espE* and *espF* have been suggested to influence biofilm formation^53^. It was recently reported that EspL is essential for *Mtb*H37Rv virulence in stabilizing the levels of EspE, EspF and EspH^54^. Whether the absence of three of these proteins has an impact on virulence for *Mmuc*- and *Mneo*-clade members remains to be investigated. Comparison of ESX-1 gene transcripts in *Mmuc*^T^ and *Mmar*^rfp^ revealed that while the mRNA levels are higher in exponential phase for the majority of the genes in *Mmuc*^T^, including the major virulence factors *esxA* (ESAT6) and *esxB* (CFP10), they increase in *Mmar*^rfp^ stationary cells (Fig. 6b). Together this might indicate differences in the regulation of these and other ESX-1 genes in the RGM *Mmuc*^T^ and SGM *Mmar*^rfp^. Moreover, the *espACD* operon suggested to be a pathogenicity-associated genomic island is present in a number of SGM pathogens^46^. In *Mtb*, export of EsxA and EsxB is suggested to be co-dependent on EspA and EspC, where EspC is localized on the bacterial surface^46,55^. In *Mmar*^rfp^, the level of the *espACD* transcript is > five-fold (log_2_) higher in stationary phase. Albeit, *espACD* is absent in *Mmuc*- and *Mneo*-clade members, *espA* and *espD* paralogs were predicted to be present in *Mmuc*-clade members and in three *Mneo* strains while *espC* appears to be missing. For the two *espA* paralogs in *Mmuc*^T^, we detected modestly higher mRNA levels in exponentially growing cells while the levels for the three *espD* paralogs were similar (if anything slightly higher in exponential phase; not shown). Together this raises questions about the function of the *espA* and *espD* paralogs and the mechanism of secretion of ESAT6 and CFP10 in *Mmuc*- and *Mneo*-clade members. In contrast ESX-3 gene transcripts were higher in exponentially growing *Mmuc*^T^ and *Mmar*^rfp^ cells. ESX-3 has a role in iron acquisition and virulence^56,57^ and hence, our findings reflect the demand of iron in growing mycobacterial cells.

### Rough and smooth colony morphotypes

Isolates of various mycobacteria such as *Mycobacterium abscessus*, *Mycobacterium salmoniphilum* and *Mmar* can form smooth and rough colony morphotypes (CM) when grown on solid media^24,52,58^. We also note that *Mycobacterium canettii* with its unusual CM is referred to as the “smooth tubercle bacilli”^59,60^. However, smooth morphotypes for certain SGM, including *M. canettii*, are due to the production of lipooligosaccharides and differs from RGM^61^. Analysis of in particular *M. abscessus*, *Msmeg* and *Mycobacterium avium* strains reveals that the smooth and rough CM are related to genes involved in generating glycopeptidolipids (GPL locus), which are exposed on the cell surface. Mutations or deletion of genes within the GPL locus can result in transition from a smooth (S) to a rough (R) CM, where mycobacteria with rough CM appear to be more virulent^24,52^. *Mmuc*^T^ is highly mucoid when grown on solid media^3,5^ and surveying the GPL locus shows that several of the genes are absent in these members of the *Mmuc*- and *Mneo*-clades. In keeping with this, *Mmuc*^T^ forms R colonies when grown on 7H10 media. In contrast, *Mpho*^T^, *Maub*^T^ and *Mcos*^T^ forms S colonies while in *Mneo*^T^ cultures we detected both R and S colonies (Fig. S8). Therefore, this indicates that factors other than genes in the GPL locus influence CM and we cannot exclude that gene transcription has a role. Considering the mucoid growth of *Mmuc* strains these findings suggest that it is not simply related to the GPL locus.

### Sigma and anti-sigma factors

As other mycobacteria *Mmuc*- and *Mneo*-clade members encode for several ECF σ-factors in addition to SigA, SigB and SigF^18,37,38,58,62^. Phylogeny suggested that these cluster into distinct groups. However, they do not cluster into groups in accordance with their annotation. Hence, this emphasizes the importance to generate phylogenetic trees in order to understand the phylogenetic relationship and evolutionary history of σ-factors and anti-σ factors. This relates also to other factors such as STPKs (*pkn* genes; Fig. S4i). More specifically, given that several ECF σ-factors are closely linked to an anti-σ factor gene [*rsbW*, *rsdA*, *rskA* (*A*1 and *A*2), *rsmA*, *rslA*, *rshA*] one important question is whether these anti-σ factors also interact with other σ-factors. Therefore, to understand and study if any of the known anti-σ factors also regulate the activity of another σ-factor information about the phylogenetic relationship of both the σ- and anti-σ factors is important. For example, previously it was reported that the *Escherichia coli* Rsd anti-σ factor interacts with the house keeping σ-factor, Sig70, and interferes with its activity^63–65^. We also note that the putative anti-σ^C^ factor (*rscA*) is not positioned close to *sigC* in *Mtb*^37,43^ indicating that anti-σ factor genes do not necessarily have to be part of the same transcriptional unit as the corresponding σ-factor gene. However, in *Mmar*^T^ and *Mmuc*-clade members the putative *rscA* is located close to *sigC* with one and four (or five) genes in between, respectively (this report; Pettersson *et al*., unpublished). *Mneo*-clade members lack *sigC* and *rscA* and relative to *Mmuc*-clade members they also lack nearby genes (Fig. S4f). Given that mycobacteria that are phylogenetically closer to the mycobacterial ancestor carry *sigC* and *rscA* (unpublished data) might indicate that the *Mneo*-clade members lost *sigC* and *rscA* after they diverged from the *Mmuc*-clade.

We recently reported variations of σ-factor mRNA levels in *Mmar*^rfp^ that depended on growth conditions with *sigB* and *sigE* mRNA levels dominating in stationary phase^18^. Although *sigB* mRNA dominates in *Mmuc*^T^ stationary cells, *sigD* and *sigL*1 levels are higher than the *sigE* homolog, *sigE*1. Together, this is in accordance with the notion that SigB is involved in general stress response in mycobacteria and that this likely also apply to SigE^18,38,65,66^. Apart from that the two *sigL* mRNAs increased significantly in stationary phase the levels for the ECFs *sigW*1, *sigW*2, *cnrH*1 and *cnrH*2 were higher. The *sigW* genes were annotated on the basis of *sigW* present in *Bacillus subtilis*, which have been suggested to be involved in mediating resistance to certain antibiotics, *e.g*. fosfomycin^67^. The *cnrH* genes were predicted as homologs of *cnrH* present in *Cupriavidus metallidurans* CH34 where it is part of a circuit regulating resistance to metals, in particular nickel^68,69^. Together this raises the possibility that stationary *Mmuc*^T^ cells are prepared to “face” exposure to antibiotics such as fosfomycin and nisin as well as nickel. Moreover, the *Mmuc*^T^ *sigD* mRNA level increased in stationary phase, which is in contrast to *Mtb* and *Mmar* where it is lower^18,70,71^. For *Mtb*, however, higher level of SigD mRNA in late stage of “exponential” growth has been reported^72^. Nevertheless, in *Mtb* SigD has been discussed to affect various stages during infection such as replication and cell division^73,74^ and to be involved in the control of ribosome-associated genes^75^. On the basis of these findings it is clear that in order to get a deeper understanding of the role of SigD (as well as the role of other *Mmuc*^T^ sigma factors), and whether there is a difference between SGM and RGM, warrants further studies. Interestingly, a functional similarity between SigW in *B. subtilis* and SigD in *Mtb* has been discussed^75^. In this context, we note that *Mneo* SigD is suggested to act as a negative regulator in phytosterol metabolism^76^.

### Antibiotic resistance – rifampin and isoniazid

The RNA polymerase (RNAP) is the target for one of the first-line anti-TB drugs, rifampin, while another first line drug, isoniazid, interferes with the building of the cell wall. Mutations in *rpoB* (RNAP β-subunit) and *katG* can result in resistance to rifampin (RifR) and isoniazid, respectively^25,77,78^ (https://tbdreamdb.ki.se/), and *Mmuc*- and *Mneo*-clade members are resistant to both these antibiotics^5,79^ (see introduction). For rifampin, we were unable to detect changes in any of the antibiotic resistance “hot spot” positions in *rpoB* that could be the reason to their natural resistance. Two other genes, *rbpA* and *arr*, have been reported to influence resistance. RbpA corresponds to an essential RNAP binding protein in mycobacteria^80–82^ while Arr is a rifampin ADP-ribosyltransferase, which catalyzes rifampin ribosylation^82^ (and Refs therein). *Mneo*-clade members have two copies of *rbpA* while for the *Mmuc*-clade members only one was predicted (Table S4a). For *arr*, the *Mmuc* strains all have two copies (MUCO_DSM_01098 and MUCO_DSM_04701) while the other mycobacteria carry one copy with the exception of the *Mneo* strains which appear to lack *arr* (Table S4b). The two *arr* are expressed both in exponential and stationary phase *Mmuc*^T^ cells. However, the mRNA level for MUCO_DSM_01098 is three times higher in exponentially growing cells than MUCO_DSM_04701. But, in stationary phase their mRNA levels are roughly equal (Fig. S7).

For isoniazid, we detected amino acid substitutions at several positions in *katG* and *inhA* where mutations have been reported to lead to resistance. For example, all *Mmuc*- and *Mneo*-clade members carry valine at 139 in *katG* while isoniazid sensitive *Mtb* has alanine at this position and mutation to valine result in isoniazid resistance^25,78^.

Together these observations might give indications as to why *Mmuc*- and *Mneo*-clade members show resistance to rifampin and isoniazid.

### Concluding remark

Our present findings together with our recent report where we analyzed the *Mmuc*- and *Mneo*-clade members focusing on tRNAs and non-coding RNAs^7^ provide insight into the biology of these two groups of rapid growing and opportunistic NTM pathogens. As such this knowledge will be useful to treat infections caused by these and other mycobacteria as well as to identify the species causing the infection. This is exemplified by the finding that an isolate classified as *Mmuc* (*Mmuc*^LZSF01^) in fact should be considered as a *Mpho* strain^7^.

## Methods

### Strains and genomes

For description of *M. mucogenicum* DSM44124 (*Mmuc*^T^), *M. phocaicum* DSM45104 (*Mpho*^T^), *M. aubagnense* DSM45150 (*Maub*^T^), *M. neoaurum* DSM44074 (*Mneo*^T^) and *M. cosmeticum* DSM44829 (*Mcos*^T^), the other *Mmuc*- and *Mneo*-clade members and *Mycobacterium* sp. URHB0044 genomes (in total 17 genomes) see Table S1. The genomes were previously deposited at NCBI under the Bioproject: PRJNA429429, see Behra *et al*.^7^.

### Genome annotation, functional classification and core genes

Genome annotation and coding sequences (CDS) were predicted using the PROKKA software (version 1.0.9)^83^. For functional classification, the predicted (PROKKA and NCBI annotated) CDS were subjected to BLASTp against the RAST predicted CDS followed by mapping to the RAST subsystem database (http://rast.nmpdr.org/, last accessed May 5, 2015)^84^ using the BLAST approach^85^.

Core genes were identified as previously reported^7^.

### Horizontal gene transfer – HGT

Putative horizontal gene transfer (HGT) were identified using the HGTector tool v1.9^86^. The prediction of HGT genes is based on the combination of BLAST search method, and the NCBI taxonomic hierarchical classification. For the BLAST search, we used the NCBI nr-database (Uppmax resource, Uppsala University, as of Sep 2015), the NCBI-BLAST version 2.2.30+ and the tool BLASTp search setting the e-value to 1e-100.

For the NCBI taxonomic classification we chose “self = *Mycobacterium*” (taxonomic_id 1763) and “close = *Actinomycetales*” (taxonomic_id 2037), where the group “distal = all other organisms except from the list of “self” and “close” groups (as of NCBI taxonomy on Sep 2015; Ref of NCBI taxonomy)^86^. The common and unique HGT genes were obtained using the PanOCTv1.9 pipeline^87^, the NCBI-BLAST (ver 2.2.30+) tool, and the tool BLASTp with a minimum percentage identity 45% and query coverage 70%. As a complement, we performed a Mann-Whitney-Wilcoxon test (in R ver 3.2.2, 2015-08-14 on platform x86_64-pc-linux-gnu) for GC content of all protein CDS and GC content of putative HGT genes.

### Prediction of virulence factor genes (VFDB)

Virulence factor genes were predicted using the tool VFanalyzer, webserver available at the Virulence Factor Data Base, VFDB^88,89^. We initially used protein CDS as input to the VFanalyzer and as reference we included *Mtb*H37Rv, *Msmeg*MC2-155, *Mmar* M strain, *M. ulcerans* AGY99, and *M. avium partuberculosis* K10 available from the VFDB database. The single XLS data files were combined and the so obtained data file were used for presentation of the data using the R interface (ggplot2 package)^90^.

For the detailed analysis of sigma factor, STPK, ESX, Mce and GPL locus genes we used *Mtb*H37Rv, *Msmeg* (and *M. abscessus*, *M. avium*) and the VFDB XLS reference dataset (see above) as references. For identification of orthologous genes we used PanOCT and pair wise analysis using the reciprocal BLASTp hit followed by verifying the corresponding protein using the SMARTdb database for protein architecture^91^.

### Phylogenetic analysis

Phylogenetic trees for sigma factor, anti-sigma factor and STPK proteins were generated based on the alignment of the respective amino acid sequences using the MAFFT (version 7.147b) software^92^. The so obtained MAFFT aligned multiple sequences were computed using the FastTree tool with 1000 cycles of bootstrapping and run with the default settings: default run infers approximately-maximum-likelihood phylogenetic trees from alignments of protein sequences, using the model Jones-Taylor-Thorton + CAT models of amino acid sequences^93^. Figures were generated using the iTOL tool webserver^94^.

The rooting of sigma factor phylogeny was set at the node level of SigA, SigB and SigF. For the cognate sigma/anti-sigma factor phylogeny SigF and RsbW were set as roots while for the STPKs we used the PknG protein.

### RNA extraction, RNA sequencing and analysis

RNASeq analysis was performed as described in detail elsewhere^18^. Briefly, *Mmuc*^T^ and *Mmar*^rfp^ (biological duplicates) were grown in 7H9 media at 37 °C and 30 °C, respectively, and total RNA was extracted from exponentially growing and stationary phase cells. The RNA was extracted using Trizol and a bead beater, DNase treated and submitted for RNA sequencing at the SNP@SEQ Technology Platform at Uppsala University (HiSeq 2000 Illumina platform).

For the *Mmuc*^T^, the RNASeq data sets (*i.e*. number of reads) were mapped to the reference complete genome by building the index using bowtie2 v2-2-4^95^ and followed by alignment with the tool Tophat v2.0.13^96^. From the aligned BAM files the read-counts were generated using the HTseq v0.9.1^97^ and normalization, differential expression analysis was performed by using the Deseq.2 package, which gives p + adj values, *i.e*. statistic significance^98^. With respect to *Mmar*^rfp^ the RNASeq data was generated as described by Pettersson *et al*.^18^.

### Ethics statement

All methods were carried out in accordance with relevant guidelines and regulations.

## Supplementary information

Supplementary Information

Supplementary Table S1

Supplementary Table S2

Supplementary Table S3

Supplementary Table S4
