# Supplementary material for: Insight into the biology of Mycobacterium mucogenicum and Mycobacterium neoaurum clade members
Source: Sci Rep. 2019 Dec 17;9:19259. doi: 10.1038/s41598-019-55464-5 (PMC6917791; doi:10.1038/s41598-019-55464-5)
Supplement: Supplementary file 1 — Supplementary Information [file 41598_2019_55464_MOESM1_ESM.pdf]

Supplementary information

Insight into the biology of *Mycobacterium mucogenicum* and *Mycobacterium neoaurum* clade members

Phani R. K. Behra, B. M. Fredrik Pettersson, Malavika Ramesh,

Santanu Dasgupta and Leif A. Kirsebom*

Department of Cell and Molecular Biology

Box 596, Biomedical Centre

SE-751 24 Uppsala, Sweden

*Corresponding author

Tel no +46 18 471 4068

Fax no +46 18 53 03 96

Email Leif.Kirsebom@icm.uu.se

Supplementary information

Supplementary Table S1: Summary of mycobacteria, genome accession numbers and description of their genomes focusing on number of: coding determining sequences (CDS), virulence factors and predicted horizontally transferred genes.

Supplementary Table S2 a-p: Compilation and analysis of predicted horizontally transferred genes in *Mmuc*- and *Mneo*-clade members.

Supplementary Table S3 a-g: Compilation of:

(a) predicted virulence factor genes in *Mmuc*- and *Mneo*-clade members,

(b) predicted MCE genes in *Mmuc*- and *Mneo*-clade members,

(c) predicted ESX genes in *Mmuc*- and *Mneo*-clade members,

(d) predicted sigma factor genes in *Mmuc*- and *Mneo*-clade members,

(e) predicted STPK genes in *Mmuc*- and *Mneo*-clade members,

(f) genes related to the glycopeptidolipids (GPL) locus in selected mycobacteria,

(g) predicted anti-sigma factor genes in *Mmuc*- and *Mneo*-clade members.

Supplementary Table S4 a, b: Compilation of:

(a) predicted *rbpA* homologs in *Mmuc*- and *Mneo*-clade members,

(b) predicted *arr* homologs in *Mmuc*- and *Mneo*-clade members.

Supplementary Figures S1-S8.

Figure S1 Phylogenetic tree encompassing Mmuc- *and* Mneo-*clade members*

Phylogenetic tree based on 2770 core genes common to all indicated *Mmuc*- and *Mneo*-clade members, for details see Behra et al. (Ref 7 main text).

Fig S1

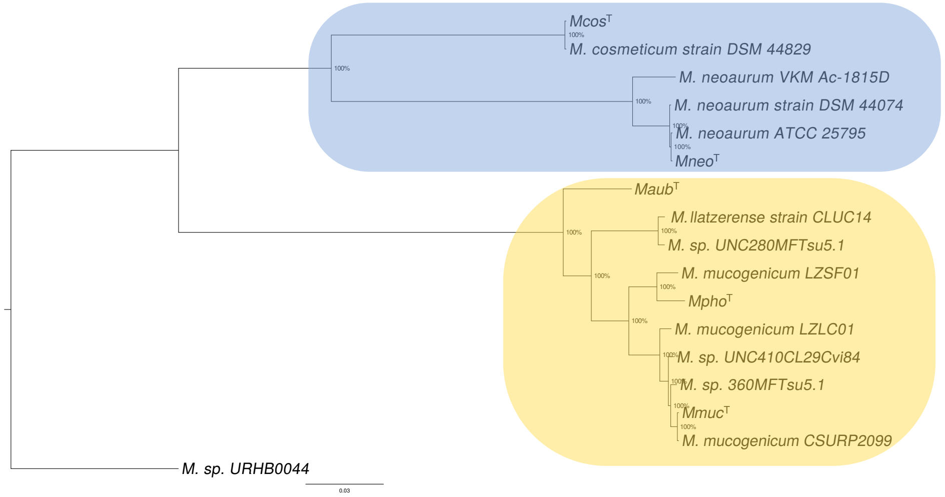

Mcos^T
100%
M. cosmeticum strain DSM 44829
100%
M. neoaurum VKM Ac-1815D
100%
M. neoaurum strain DSM 44074
100%
M. neoaurum ATCC 25795
100%
Mneo^T
Maub^T
100%
M. llatzerense strain CLUC14
100%
M. sp. UNC280MFTsu5.1
100%
M. mucogenicum LZSF01
100%
Mpho^T
100%
M. mucogenicum LZLC01
100%
M. sp. UNC410CL29Cvi84
100%
M. sp. 360MFTsu5.1
100%
Mmuc^T
100%
M. mucogenicum CSURP2099
M. sp. URHB0044
0.03

Figure S2 Functional classification of CDS in Mmuc- *and* Mneo-*clade members*

(a) Functional classification of CDS *Mmuc*- and *Mneo*-clade members, *Msp.* URHB0044, *Msmeg*, *Mtb*H37Rv and *Mmar*^{T} where *Msmeg* and *Mtb*H37Rv were used as references.

(b) Functional classification of core genes specific to *Mmuc*- and *Mneo*-clade members as indicated.

(c) Carbohydrates - subcategory functional classification of core genes specific to *Mmuc*- and *Mneo*-clade members as indicated.

Fig S2

a)

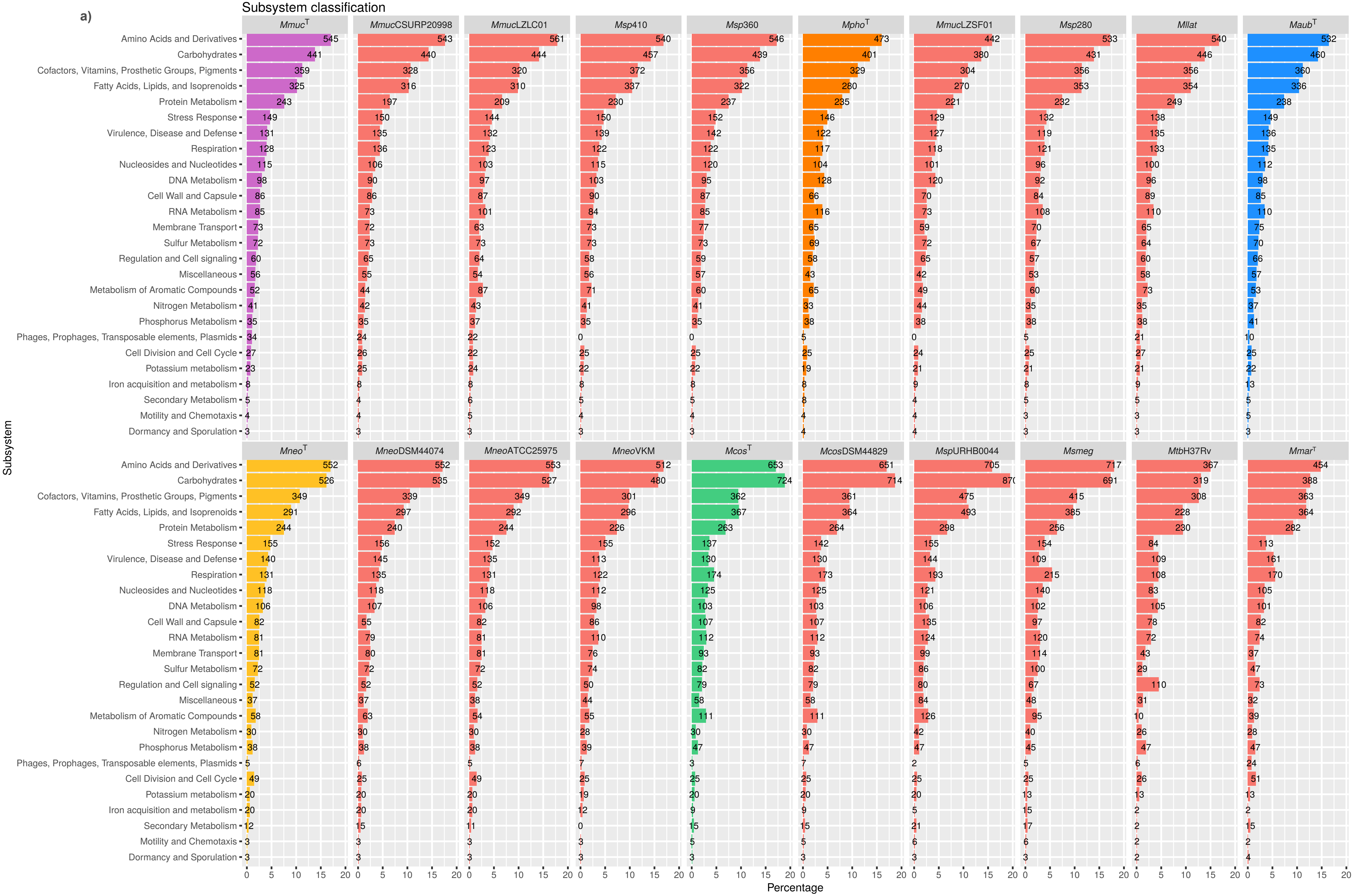
Subsystem classification
MmucT
MmucCSURP20998
MmucLZLC01
Msp410
Msp360
MphoT
MmucLZSF01
Msp280
Mllat
MaubT
MneoT
MneoDSM44074
MneoATCC25975
MneoVKM
McosT
McosDSM44829
MspURHB0044
Msmeg
MtbH37Rv
MmarT
Amino Acids and Derivatives
Carbohydrates
Cofactors, Vitamins, Prosthetic Groups, Pigments
Fatty Acids, Lipids, and Isoprenoids
Protein Metabolism
Stress Response
Virulence, Disease and Defense
Respiration
Nucleosides and Nucleotides
DNA Metabolism
Cell Wall and Capsule
RNA Metabolism
Membrane Transport
Sulfur Metabolism
Regulation and Cell signaling
Miscellaneous
Metabolism of Aromatic Compounds
Nitrogen Metabolism
Phosphorus Metabolism
Phages, Prophages, Transposable elements, Plasmids
Cell Division and Cell Cycle
Potassium metabolism
Iron acquisition and metabolism
Secondary Metabolism
Motility and Chemotaxis
Dormancy and Sporulation
Subsystem
Percentage

Fig S2

b)

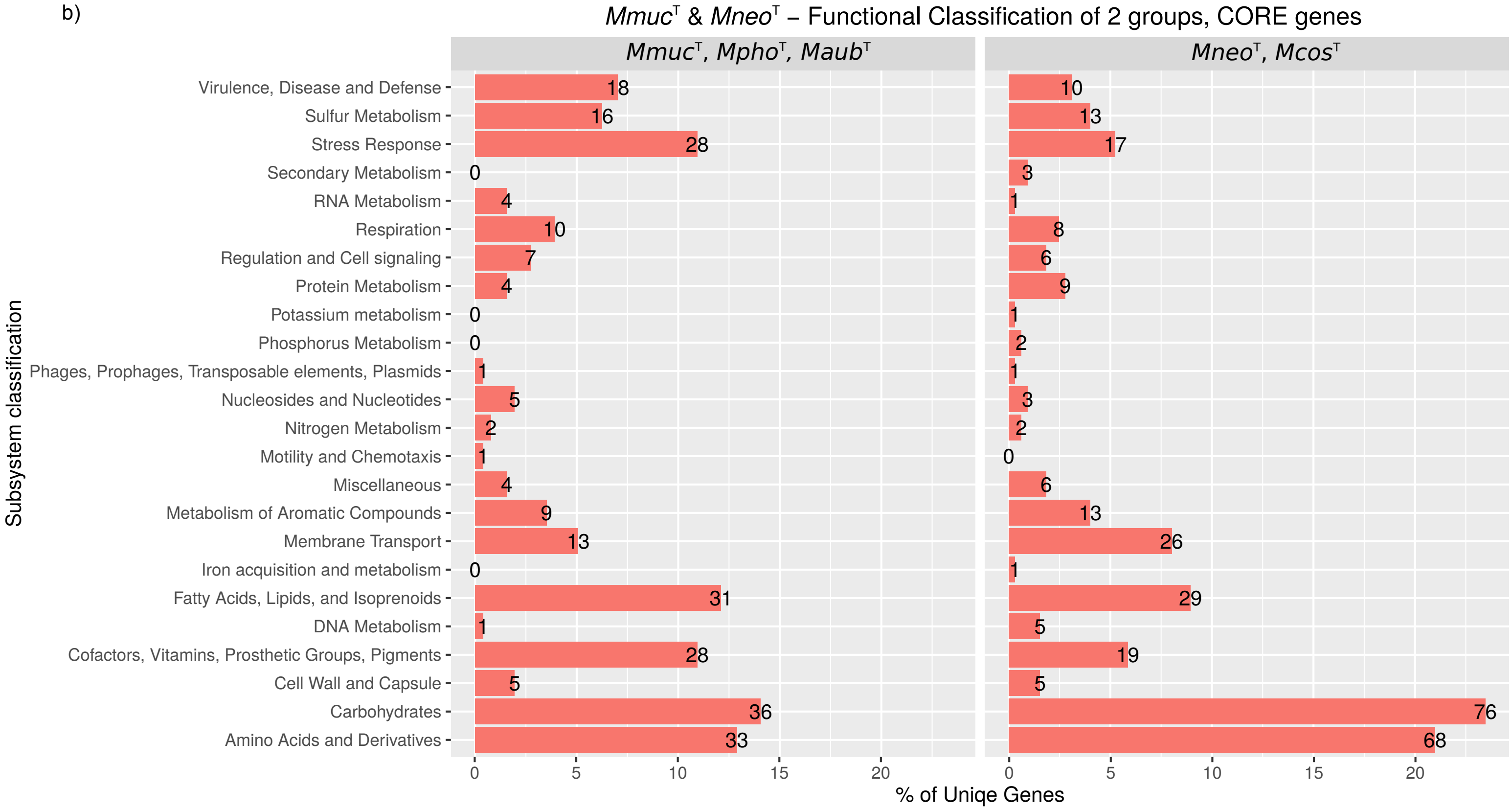
*Mmuc*ᵀ & *Mneo*ᵀ – Functional Classification of 2 groups, CORE genes
*Mmuc*ᵀ, *Mpho*ᵀ, *Maub*ᵀ
*Mneo*ᵀ, *Mcos*ᵀ
Subsystem classification
% of Uniqe Genes
Virulence, Disease and Defense
Sulfur Metabolism
Stress Response
Secondary Metabolism
RNA Metabolism
Respiration
Regulation and Cell signaling
Protein Metabolism
Potassium metabolism
Phosphorus Metabolism
Phages, Prophages, Transposable elements, Plasmids
Nucleosides and Nucleotides
Nitrogen Metabolism
Motility and Chemotaxis
Miscellaneous
Metabolism of Aromatic Compounds
Membrane Transport
Iron acquisition and metabolism
Fatty Acids, Lipids, and Isoprenoids
DNA Metabolism
Cofactors, Vitamins, Prosthetic Groups, Pigments
Cell Wall and Capsule
Carbohydrates
Amino Acids and Derivatives
18
16
28
0
4
10
7
4
0
0
1
5
2
1
4
9
13
0
31
1
28
5
36
33
10
13
17
3
1
8
6
9
1
2
1
3
2
0
6
13
26
1
29
5
19
5
76
68
0
5
10
15
20

c)

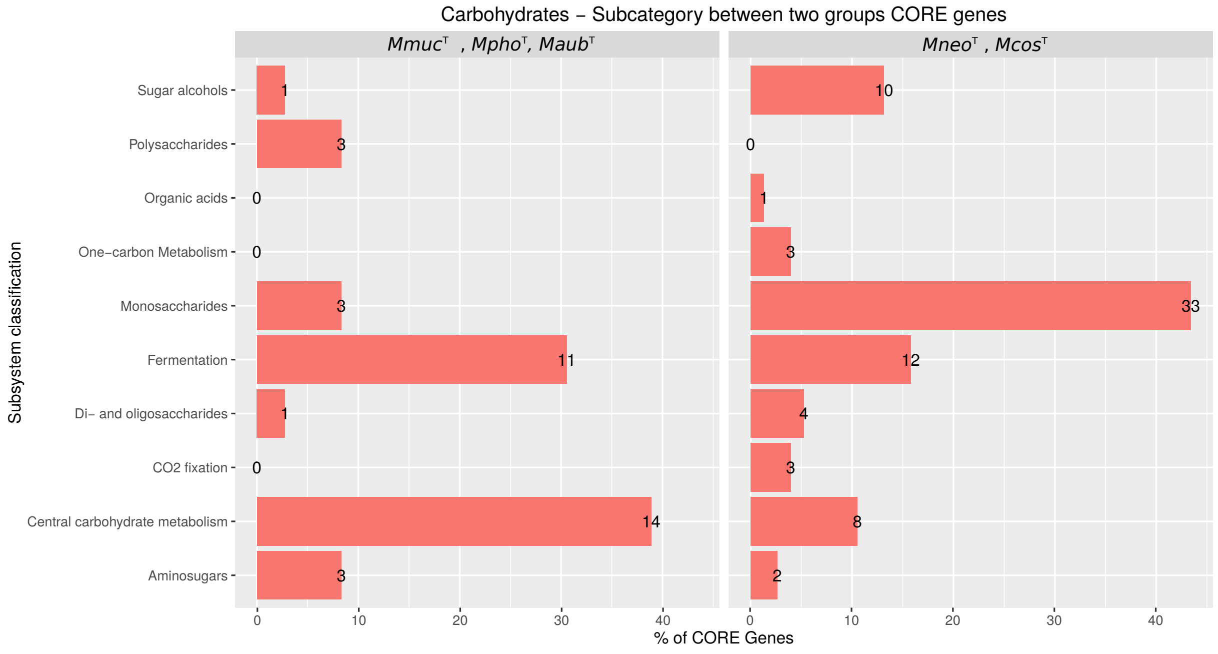
Carbohydrates – Subcategory between two groups CORE genes
*Mmuc*ᵀ , *Mpho*ᵀ, *Maub*ᵀ
*Mneo*ᵀ , *Mcos*ᵀ
Subsystem classification
% of CORE Genes
Sugar alcohols
Polysaccharides
Organic acids
One−carbon Metabolism
Monosaccharides
Fermentation
Di− and oligosaccharides
CO2 fixation
Central carbohydrate metabolism
Aminosugars
1
3
0
0
3
11
1
0
14
3
10
0
1
3
33
12
4
3
8
2
0
10
20
30
40

Figure S3 Prediction of virulence factor genes

Prediction of virulence factor related genes using the VFanalyzer tool (see Methods in the main text) in *Mmuc*- and *Mneo*-clade members, *Msp.* URHB0044, *Msmeg*, *Mtb*H37Rv and *Mmar*^{T}.

Fig S3

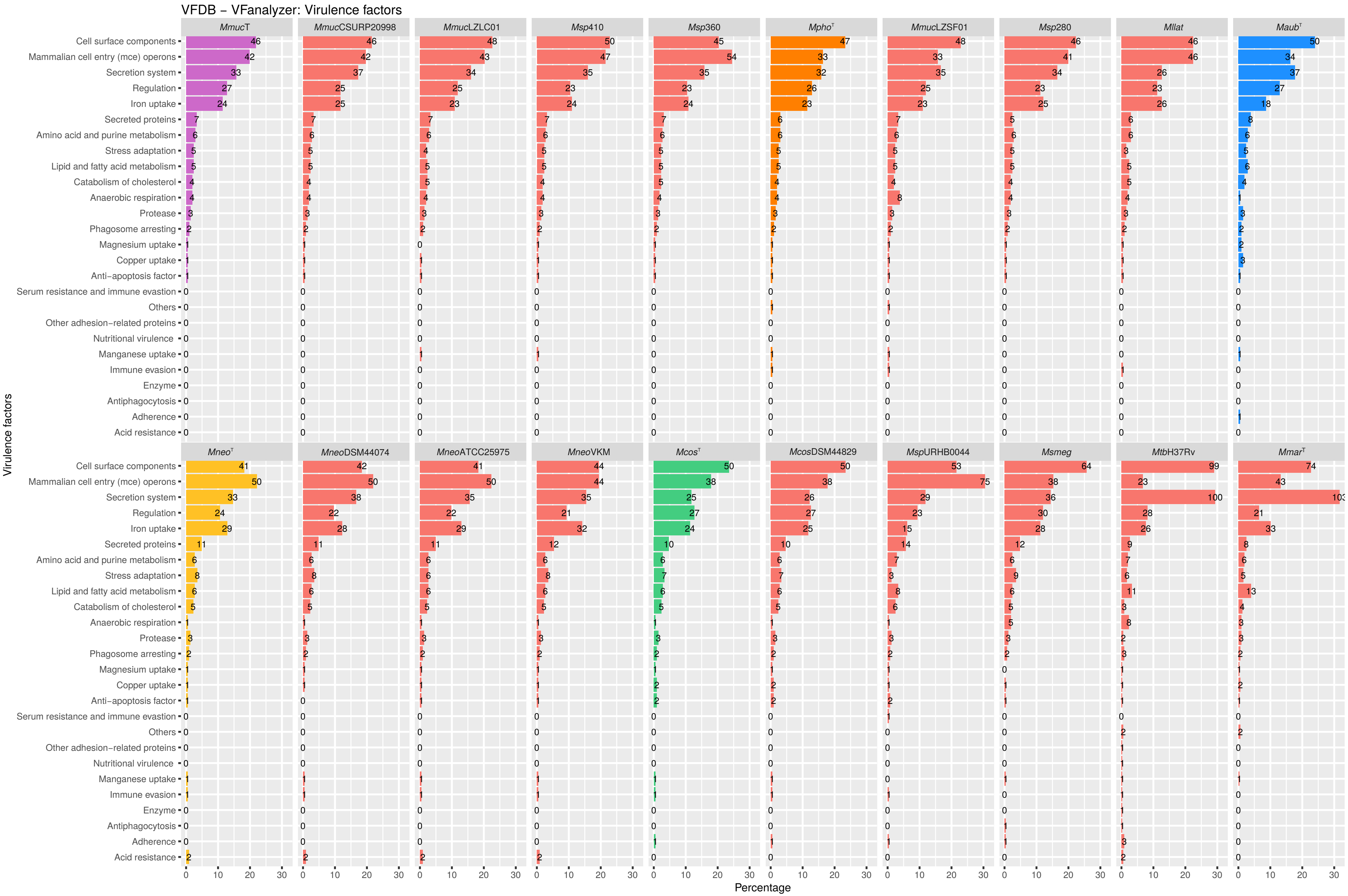
VFDB – VFanalyzer: Virulence factors
MmucT
MmucCSURP20998
MmucLZLC01
Msp410
Msp360
Mpho^T
MmucLZSF01
Msp280
Mllat
Maub^T
Mneo^T
MneoDSM44074
MneoATCC25975
MneoVKM
Mcos^T
McosDSM44829
MspURHB0044
Msmeg
MtbH37Rv
Mmar^T
Cell surface components
Mammalian cell entry (mce) operons
Secretion system
Regulation
Iron uptake
Secreted proteins
Amino acid and purine metabolism
Stress adaptation
Lipid and fatty acid metabolism
Catabolism of cholesterol
Anaerobic respiration
Protease
Phagosome arresting
Magnesium uptake
Copper uptake
Anti−apoptosis factor
Serum resistance and immune evastion
Others
Other adhesion−related proteins
Nutritional virulence
Manganese uptake
Immune evasion
Enzyme
Antiphagocytosis
Adherence
Acid resistance
Virulence factors
Percentage

Figure S4 Analysis of sigma and anti-sigma factors in Mmuc- *and* Mneo-*clade members*

(a) Circos plot showing the location of sigma factor genes on *Mmuc*^{T} and *Mmar*^{T} chromosomes.

(b) Circos plot showing the location of sigma factor genes on *Mmuc*^{T} and *Mneo*^{VKM} chromosomes.

For (a) and (b) generation of circus plots, see Krzywinski, M. et al. (2009; see below) and http://circos.ca

(c) Alignments of sigma factor gene paralogs predicted in *Mmuc*- and *Mneo*-clade members.

(d) Sigma factor phylogeny based on amino acid sequences including sigma factor genes predicted in *Mmuc*- and *Mneo*-clade members, *Msp.* URHB0044, *Msmeg*, *Mtb*H37Rv and *Mmar*^{T}. *Msmeg* and *Mtb*H37Rv were used as references.

(e) Prediction of anti-sigma factor genes among *Mmuc*- and *Mneo*-clade members, *Msp.* URHB0044, *Msmeg*, *Mtb*H37Rv and *Mmar*^{T}. *Msmeg* and *Mtb*H37Rv were used as references.

(f) Analysis of the *sigC* and *rscA* gene synteny in *Mmuc*- and *Mneo*-clade members, *Mtb*H37Rv, *Msmeg*, and *Mmar*^{T}.

Generation of gene synteny plots see Guy et al. (2010; see below) and http://genoplotr.r-forge.r-project.org/

(g and h) Sigma factor and anti-sigma factor phylogeny based on amino acid sequences, see also Fig S4d above. The trees were rooted with respect to SigF (g) and RsbW (h).

(i) STPK phylogeny based on amino acid sequences.

Extra references

Krzywinski, M. et al. Circos: An information aesthetic for comparative genomics. *Genome Res*. **19**, 1639-45 (2009).

Guy, L., Roat Kultima, J. and Andersson, S.G.E. genoPlotR: comparative gene and genome visualization in R. *Bioinformatics* **26**, 2334-35 (2010).

Fig S4

a)

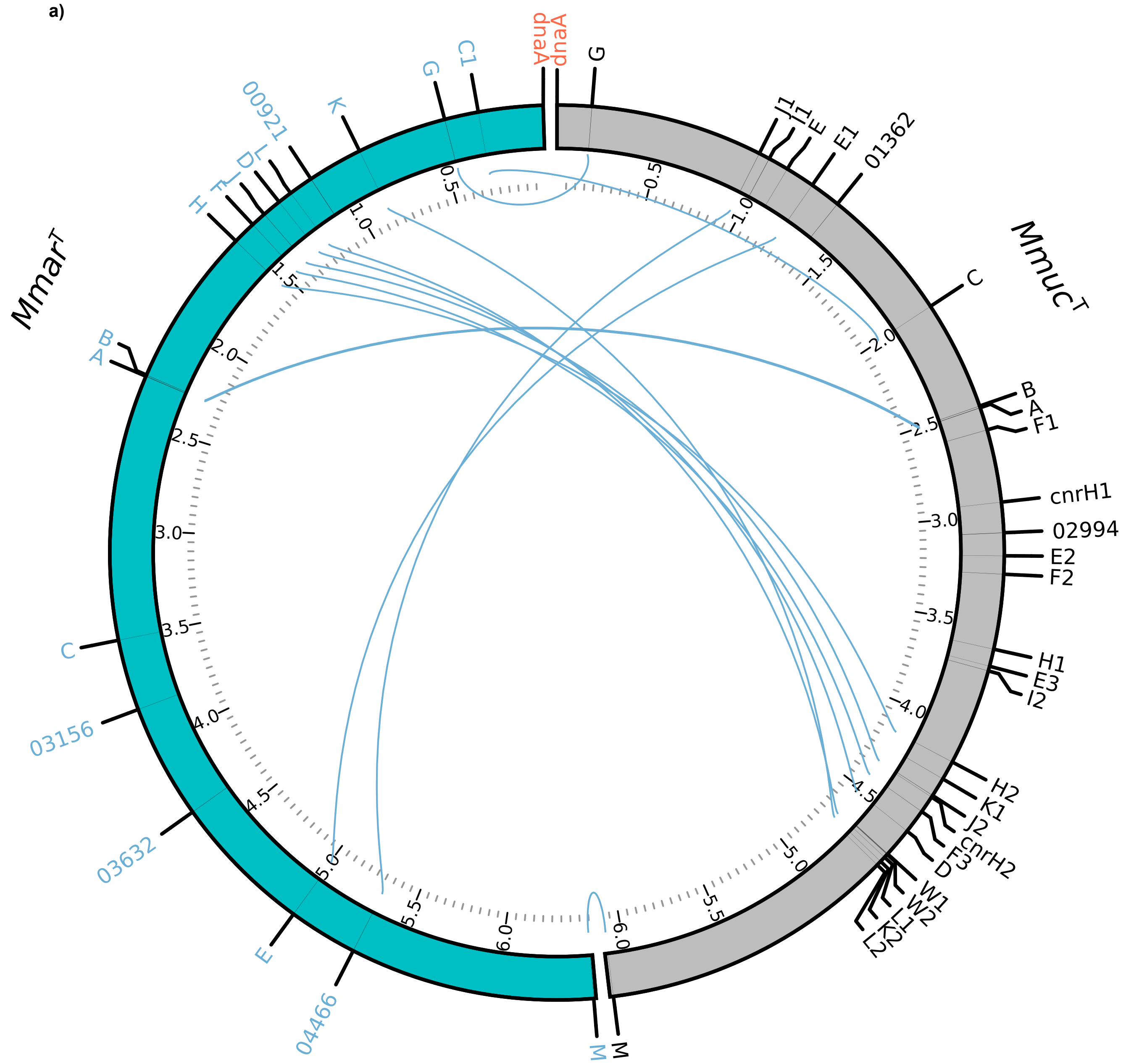

MmarT
MmucT
dnaA
dnaA
G
C1
K
00921
L
D
F
H
B
A
C
03156
03632
E
04466
M
M
G
J1
I1
E
E1
01362
C
B
A
F1
cnrH1
02994
E2
F2
H1
E3
I2
H2
K1
J2
cnrH2
F3
D
W1
W2
I
L
K2
L2

Fig S4 b)

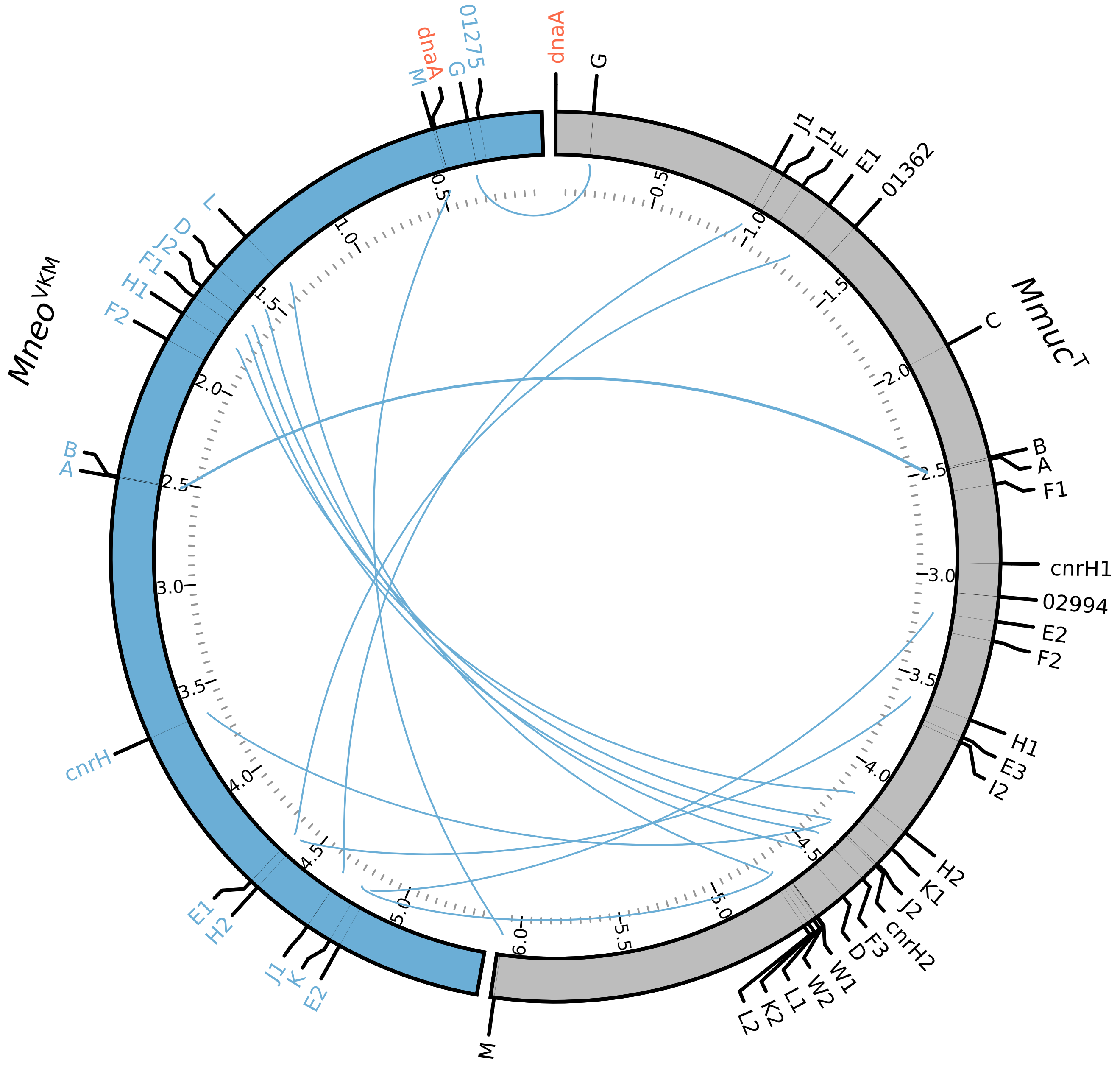

Mneo^VKM
Mmuc^T
dnaA
01275
M
G
L
D
J2
F1
H1
F2
B
A
cnrH
E1
H2
J1
K
E2
dnaA
G
J1
I1
E
E1
01362
C
B
A
F1
cnrH1
02994
E2
F2
H1
E3
I2
H2
K1
J2
cnrH2
F3
D
W1
W2
L1
K2
L2
M

Fig S4 c)

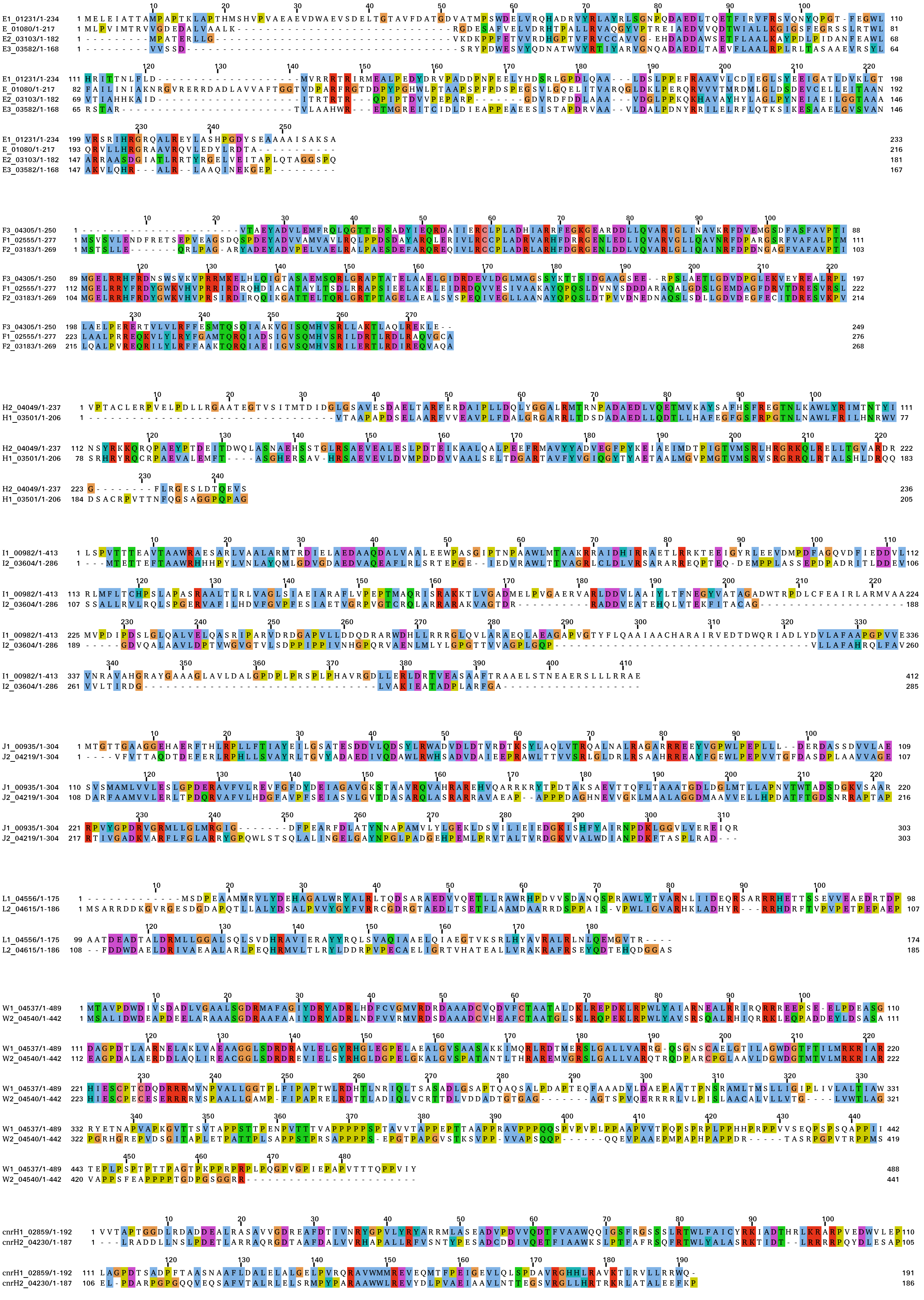
E1_01231/1-234
E_01080/1-217
E2_03103/1-182
E3_03582/1-168
F3_04305/1-250
F1_02555/1-277
F2_03183/1-269
H2_04049/1-237
H1_03501/1-206
I1_00982/1-413
I2_03604/1-286
J1_00935/1-304
J2_04219/1-304
L1_04556/1-175
L2_04615/1-186
W1_04537/1-489
W2_04540/1-442
cnrH1_02859/1-192
cnrH2_04230/1-187

Fig S4 d)

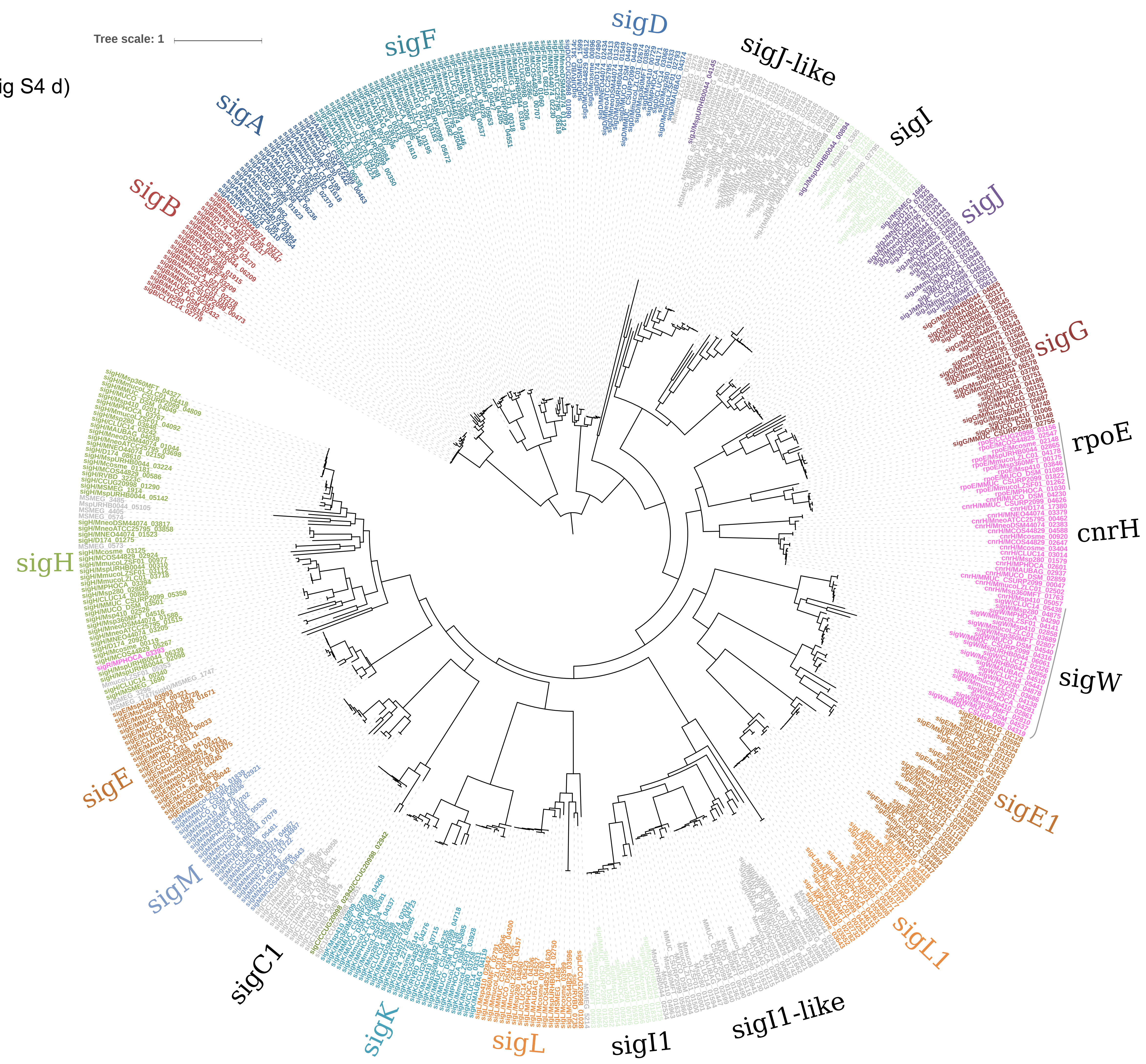

Tree scale: 1
sigD
sigF
sigJ-like
sigA
sigI
sigB
sigJ
sigG
rpoE
cnrH
sigH
sigW
sigE
sigE1
sigM
sigL1
sigC1
sigK
sigL
sigI1
sigI1-like

Fig S4 e)

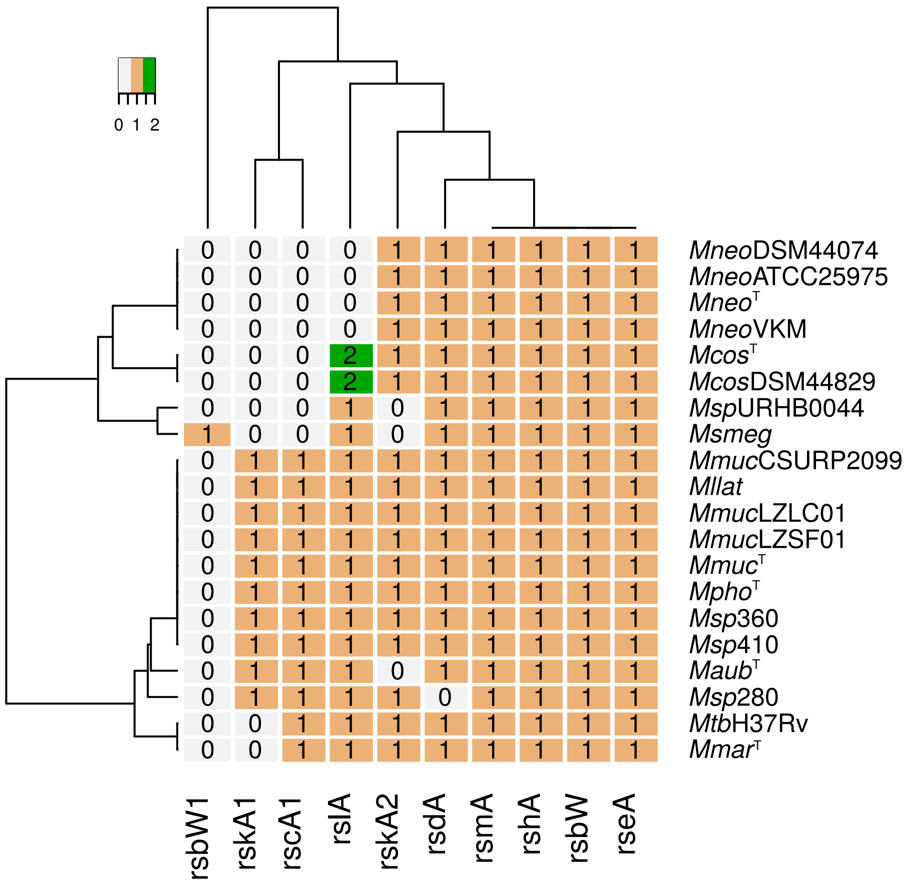
0 1 2
MneoDSM44074
MneoATCC25975
Mneo^T
MneoVKM
Mcos^T
McosDSM44829
MspURHB0044
Msmeg
MmucCSURP2099
Mllat
MmucLZLC01
MmucLZSF01
Mmuc^T
Mpho^T
Msp360
Msp410
Maub^T
Msp280
MtbH37Rv
Mmar^T
rsbW1
rskA1
rscA1
rslA
rskA2
rsdA
rsmA
rshA
rsbW
rseA

Fig S4 f)

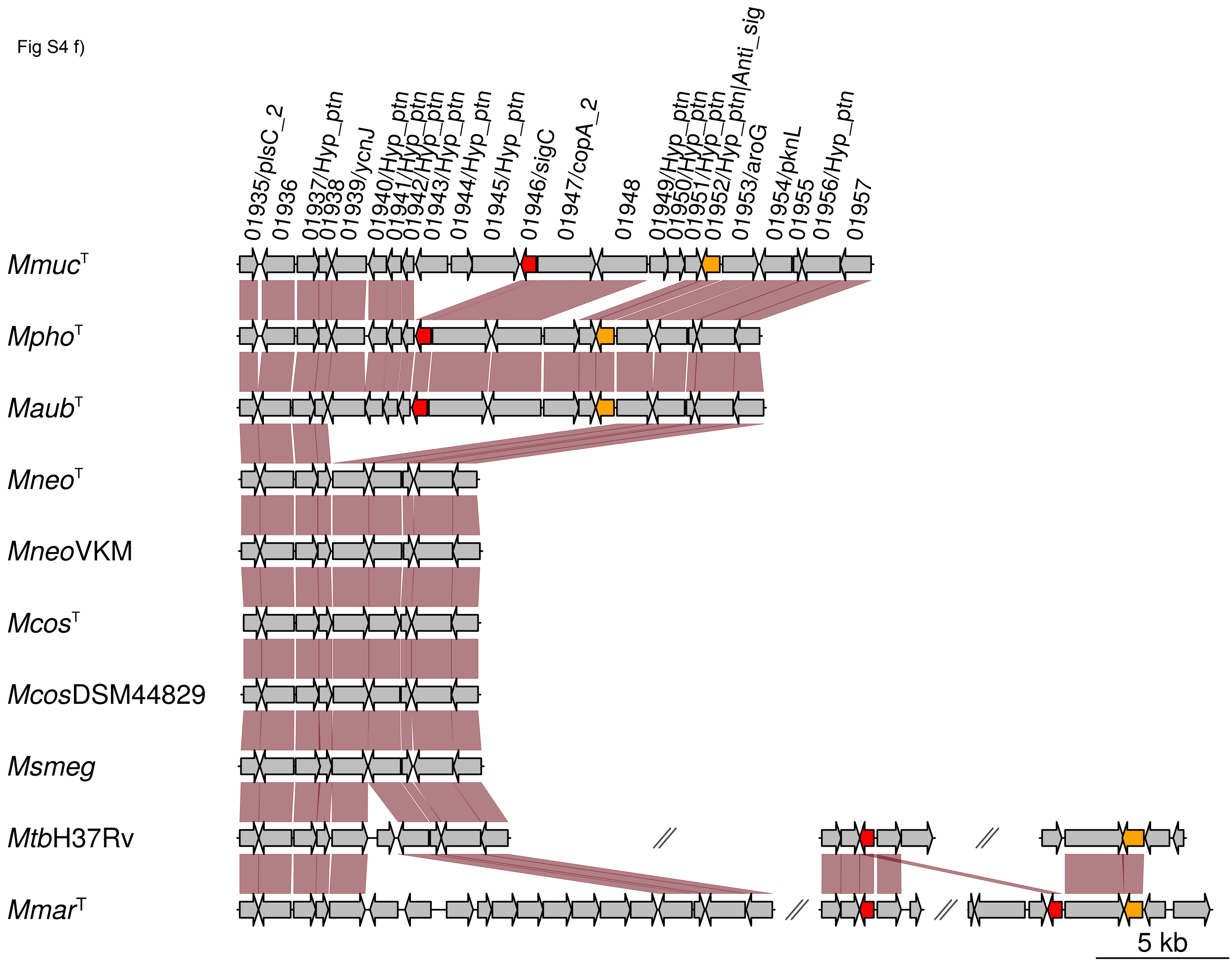
01935/plsC_2
01936
01937/Hyp_ptn
01938
01939/ycnJ
01940/Hyp_ptn
01941/Hyp_ptn
01942/Hyp_ptn
01943/Hyp_ptn
01944/Hyp_ptn
01945/Hyp_ptn
01946/sigC
01947/copA_2
01948
01949/Hyp_ptn
01950/Hyp_ptn
01951/Hyp_ptn
01952/Hyp_ptn|Anti_sig
01953/aroG
01954/pknL
01955
01956/Hyp_ptn
01957
Mmuc[T]
Mpho[T]
Maub[T]
Mneo[T]
MneoVKM
Mcos[T]
McosDSM44829
Msmeg
MtbH37Rv
Mmar[T]
5 kb

Fig S4

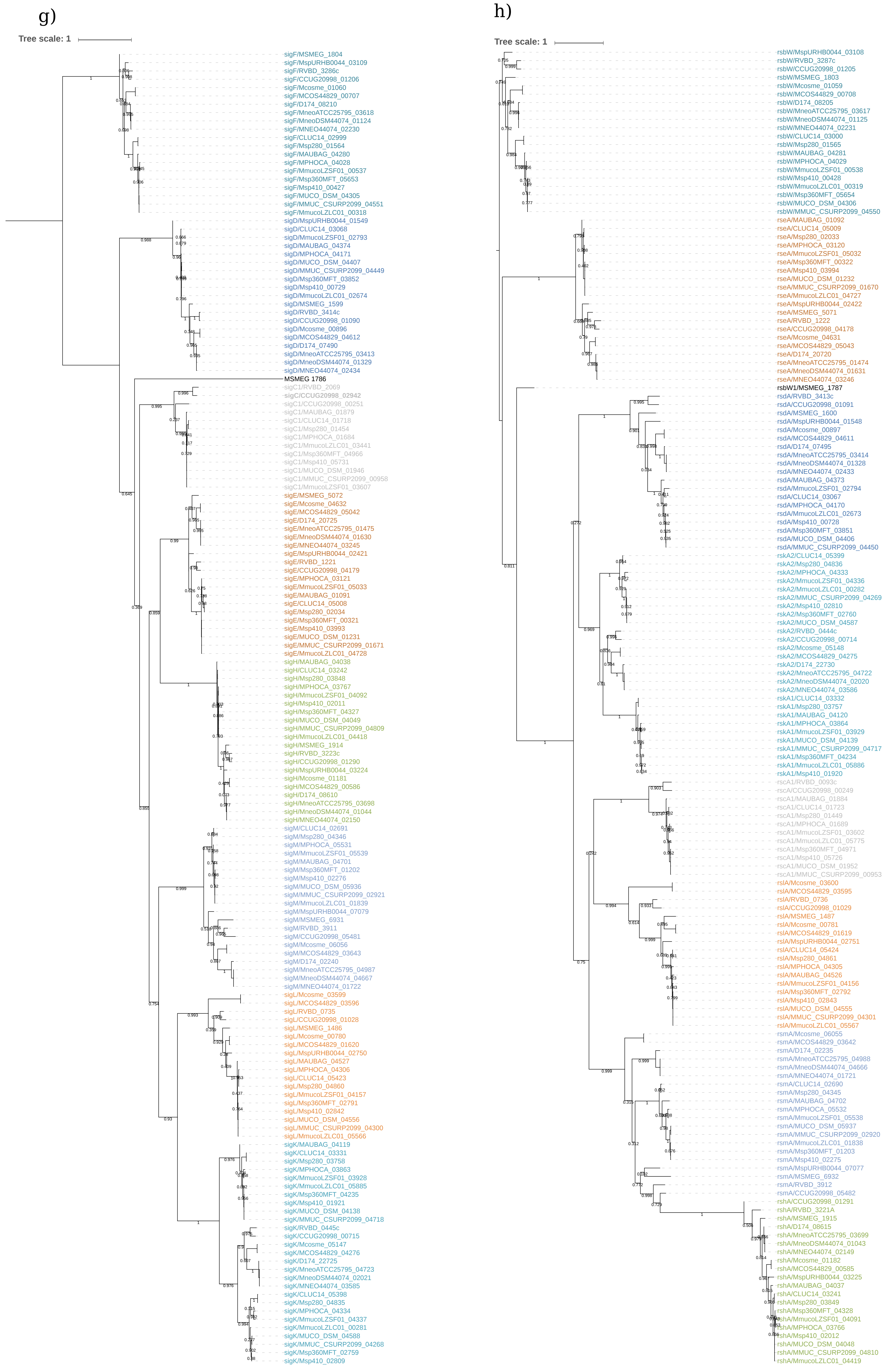

Sigma factor gene phylogeny

Anti-sigma factor gene phylogeny

Fig S4 i)

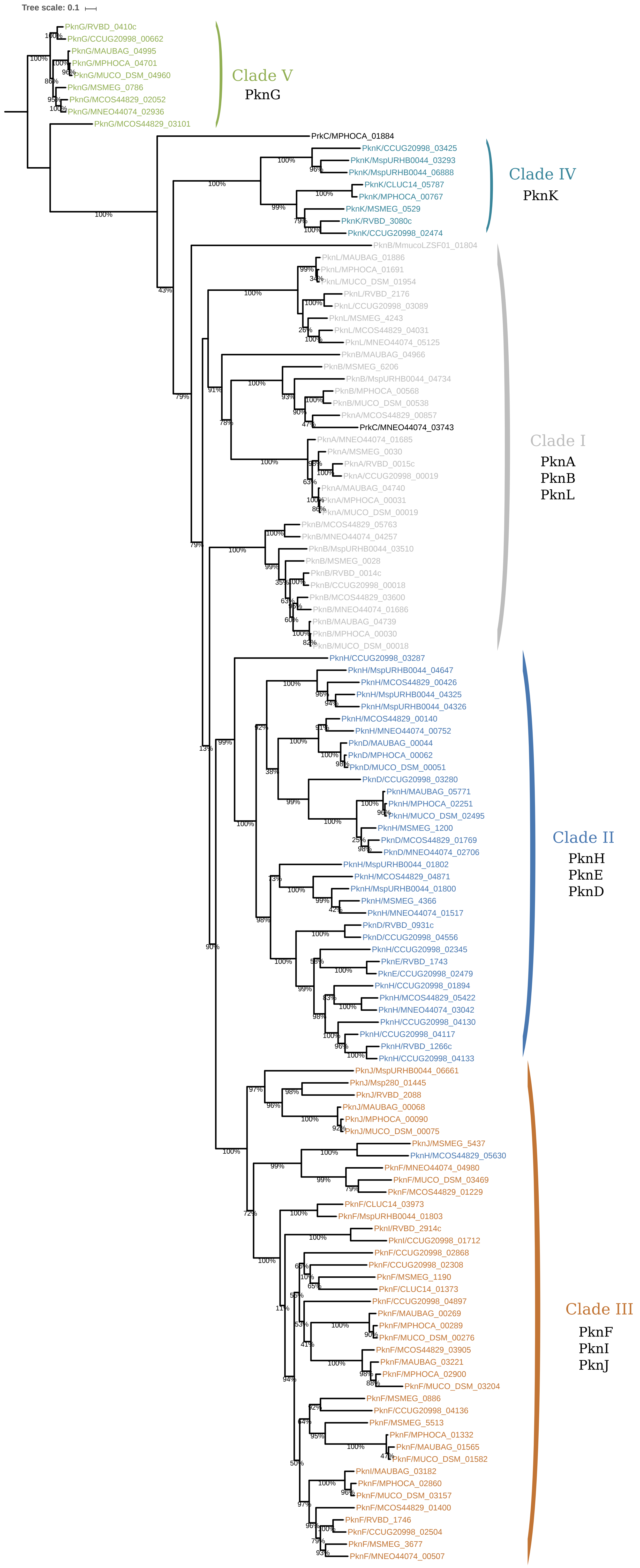

Tree scale: 0.1
PknG/RVBD_0410c
PknG/CCUG20998_00662
PknG/MAUBAG_04995
PknG/MPHOCA_04701
PknG/MUCO_DSM_04960
PknG/MSMEG_0786
PknG/MCOS44829_02052
PknG/MNEO44074_02936
PknG/MCOS44829_03101
Clade V
PknG
PrkC/MPHOCA_01884
PknK/CCUG20998_03425
PknK/MspURHB0044_03293
PknK/MspURHB0044_06888
PknK/CLUC14_05787
PknK/MPHOCA_00767
PknK/MSMEG_0529
PknK/RVBD_3080c
PknK/CCUG20998_02474
Clade IV
PknK
PknB/MmucoLZSF01_01804
PknL/MAUBAG_01886
PknL/MPHOCA_01691
PknL/MUCO_DSM_01954
PknL/RVBD_2176
PknL/CCUG20998_03089
PknL/MSMEG_4243
PknL/MCOS44829_04031
PknL/MNEO44074_05125
PknB/MAUBAG_04966
PknB/MSMEG_6206
PknB/MspURHB0044_04734
PknB/MPHOCA_00568
PknB/MUCO_DSM_00538
PknA/MCOS44829_00857
PrkC/MNEO44074_03743
PknA/MNEO44074_01685
PknA/MSMEG_0030
PknA/RVBD_0015c
PknA/CCUG20998_00019
PknA/MAUBAG_04740
PknA/MPHOCA_00031
PknA/MUCO_DSM_00019
PknB/MCOS44829_05763
PknB/MNEO44074_04257
PknB/MspURHB0044_03510
PknB/MSMEG_0028
PknB/RVBD_0014c
PknB/CCUG20998_00018
PknB/MCOS44829_03600
PknB/MNEO44074_01686
PknB/MAUBAG_04739
PknB/MPHOCA_00030
PknB/MUCO_DSM_00018
Clade I
PknA
PknB
PknL
PknH/CCUG20998_03287
PknH/MspURHB0044_04647
PknH/MCOS44829_00426
PknH/MspURHB0044_04325
PknH/MspURHB0044_04326
PknH/MCOS44829_00140
PknH/MNEO44074_00752
PknD/MAUBAG_00044
PknD/MPHOCA_00062
PknD/MUCO_DSM_00051
PknD/CCUG20998_03280
PknH/MAUBAG_05771
PknH/MPHOCA_02251
PknH/MUCO_DSM_02495
PknH/MSMEG_1200
PknD/MCOS44829_01769
PknD/MNEO44074_02706
PknH/MspURHB0044_01802
PknH/MCOS44829_04871
PknH/MspURHB0044_01800
PknH/MSMEG_4366
PknH/MNEO44074_01517
PknD/RVBD_0931c
PknD/CCUG20998_04556
PknH/CCUG20998_02345
PknE/RVBD_1743
PknE/CCUG20998_02479
PknH/CCUG20998_01894
PknH/MCOS44829_05422
PknH/MNEO44074_03042
PknH/CCUG20998_04130
PknH/CCUG20998_04117
PknH/RVBD_1266c
PknH/CCUG20998_04133
Clade II
PknH
PknE
PknD
PknJ/MspURHB0044_06661
PknJ/Msp280_01445
PknJ/RVBD_2088
PknJ/MAUBAG_00068
PknJ/MPHOCA_00090
PknJ/MUCO_DSM_00075
PknJ/MSMEG_5437
PknH/MCOS44829_05630
PknF/MNEO44074_04980
PknF/MUCO_DSM_03469
PknF/MCOS44829_01229
PknF/CLUC14_03973
PknF/MspURHB0044_01803
PknI/RVBD_2914c
PknI/CCUG20998_01712
PknF/CCUG20998_02868
PknF/CCUG20998_02308
PknF/MSMEG_1190
PknF/CLUC14_01373
PknF/CCUG20998_04897
PknF/MAUBAG_00269
PknF/MPHOCA_00289
PknF/MUCO_DSM_00276
PknF/MCOS44829_03905
PknF/MAUBAG_03221
PknF/MPHOCA_02900
PknF/MUCO_DSM_03204
PknF/MSMEG_0886
PknF/CCUG20998_04136
PknF/MSMEG_5513
PknF/MPHOCA_01332
PknF/MAUBAG_01565
PknF/MUCO_DSM_01582
PknI/MAUBAG_03182
PknF/MPHOCA_02860
PknF/MUCO_DSM_03157
PknF/MCOS44829_01400
PknF/RVBD_1746
PknF/CCUG20998_02504
PknF/MSMEG_3677
PknF/MNEO44074_00507
Clade III
PknF
PknI
PknJ

Figure S5 Analysis of sigma factor mRNA levels in exponentially growing and stationary Mmar^{T} *cells*

(a and b) Distribution profiles for *Mmar*^{rfp} (*rfp*, red fluorescent protein encoding gene, integrated into the chromosome, see Ref 18, main text) sigma factor mRNAs at different growth conditions expressed as percentage, panel (a) exponentially growing cells and panel (b) stationary cells. For details see main text.

(c) Change, expressed as $\log_2$-fold change, comparing mRNA levels in exponentially growing and stationary *Mmar*^{rfp} cells. A negative $\log_2$-value suggests that the corresponding mRNA is more abundant in exponentially growing cells while a positive value suggest higher levels in stationary cells.

Statistical significance, see Methods; *: $p< 0.05$; **: $p < 0.01$; ***: $p< 0.001$.

Fig S5

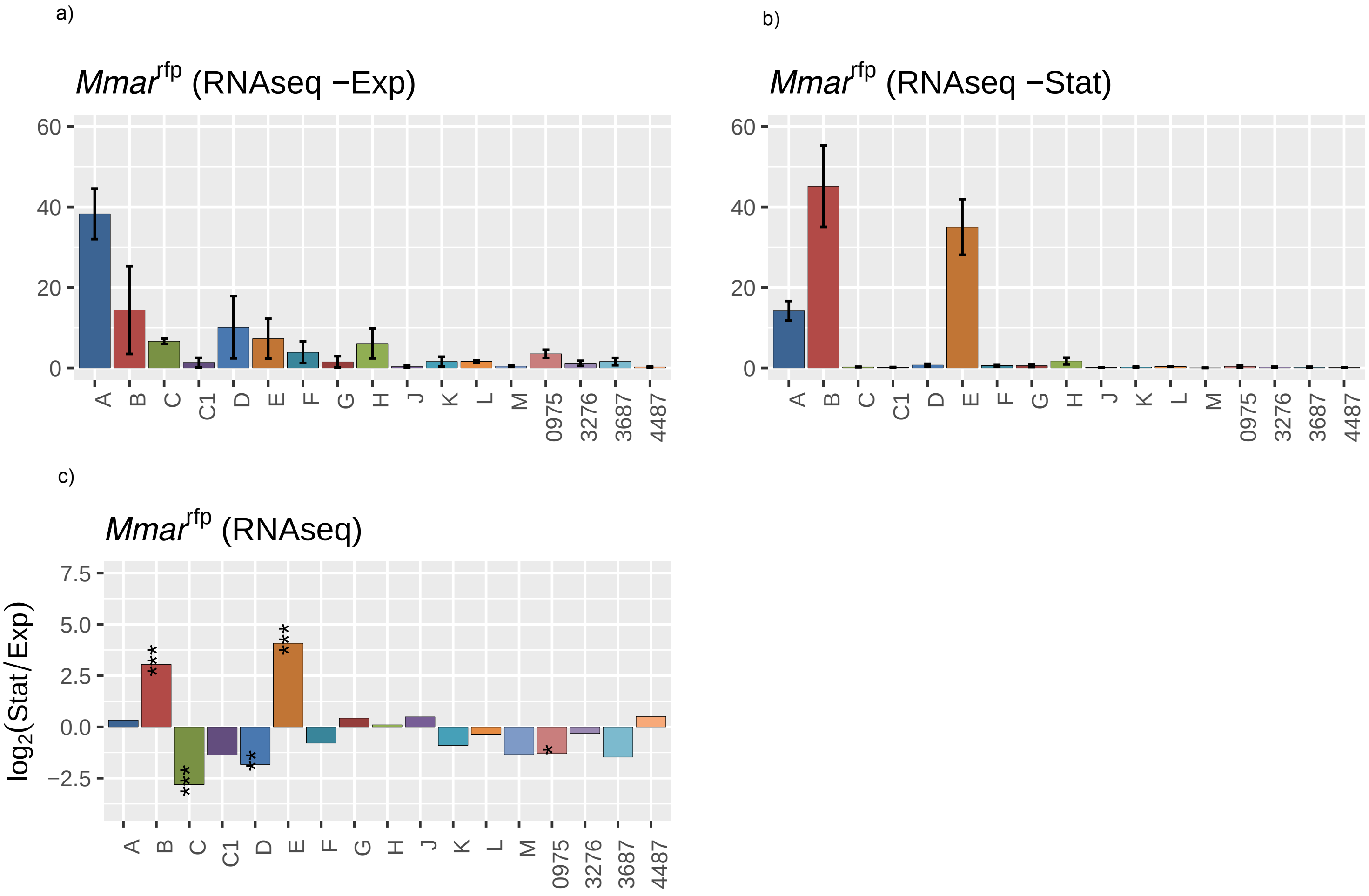
a)
Mmar rfp (RNAseq −Exp)
0
20
40
60
A B C C1 D E F G H J K L M 0975 3276 3687 4487
b)
Mmar rfp (RNAseq −Stat)
0
20
40
60
A B C C1 D E F G H J K L M 0975 3276 3687 4487
c)
Mmar rfp (RNAseq)
log2(Stat/Exp)
7.5
5.0
2.5
0.0
−2.5
***
***
***
**
*
A B C C1 D E F G H J K L M 0975 3276 3687 4487

Figure S6 Analysis of STPK, ESX and MCE mRNA levels in exponentially growing and stationary Mmuc^{T} *and* Mmar^{T} *cells as indicated*

(a and b) Distribution profiles for *Mmuc*^{T} STPK mRNA levels at different growth conditions expressed as percentage, panel (a) exponentially growing cells and panel (b) stationary cells. For details see main text.

(c and d) Distribution profiles for *Mmar*^{rfp} STPK mRNA levels at different growth conditions expressed as percentage, panel (c) exponentially growing cells and panel (d) stationary cells. For details see main text.

(e and f) Distribution profiles for *Mmuc*^{T} ESX mRNA levels at different growth conditions expressed as percentage, panel (e) exponentially growing cells and panel (f) stationary cells. For details see main text.

(g and h) Distribution profiles for *Mmuc*^{T} Mce mRNA levels at different growth conditions expressed as percentage, panel (g) exponentially growing cells and panel (h) stationary cells. For details see main text.

Statistical significance, see Methods; *: $p< 0.05$; **: $p < 0.01$; ***: $p< 0.001$.

(i) Change, expressed as $\log_2$-fold change (see g and h above), for all Mce mRNA levels comparing exponentially growing and stationary *Mmuc*^{T} cells.

Statistical significance, see Methods; *: $p< 0.05$; **: $p < 0.01$; ***: $p< 0.001$.

Fig S6)

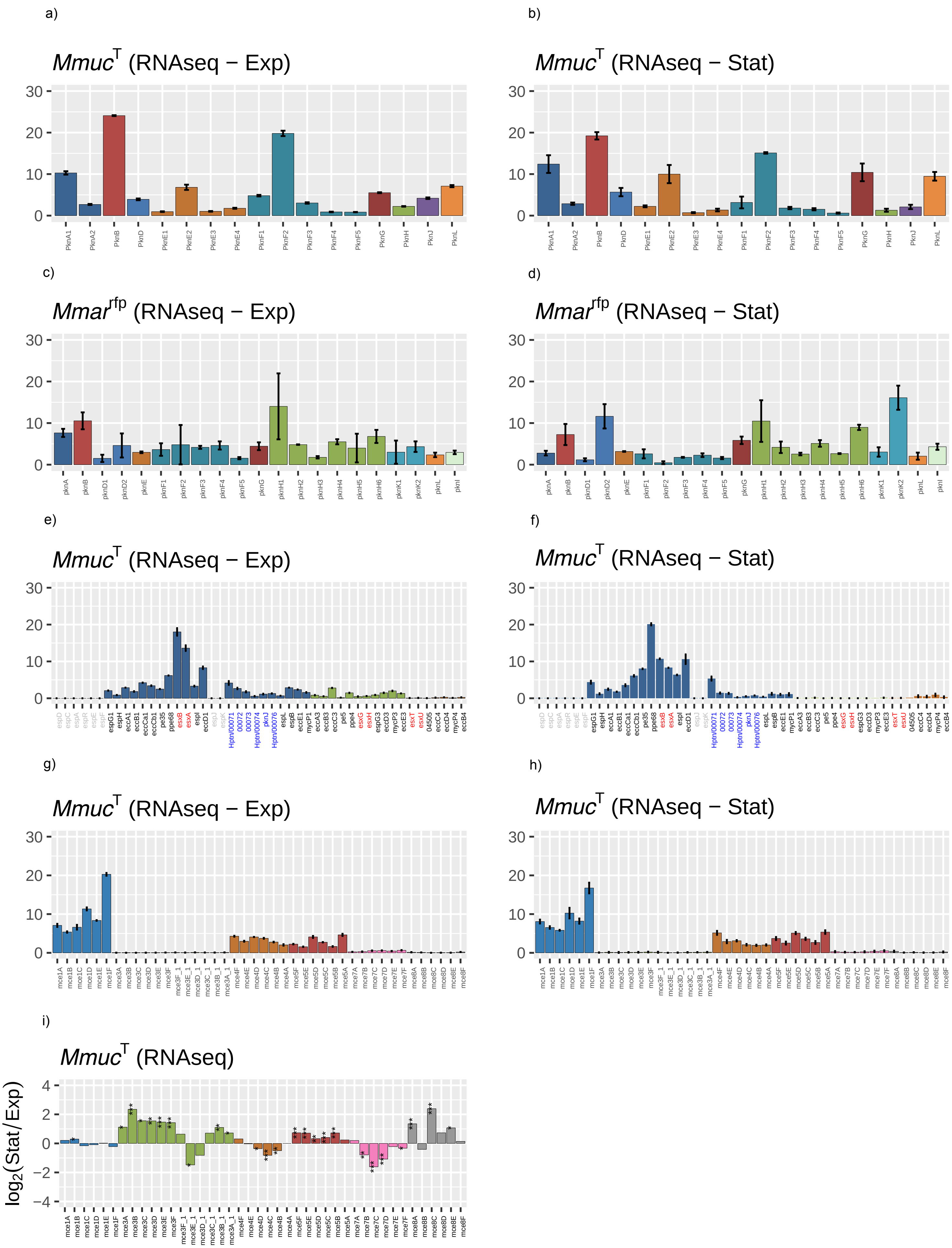

a)
Mmuc^T (RNAseq − Exp)
b)
Mmuc^T (RNAseq − Stat)
PknA1 PknA2 PknB PknD PknE1 PknE2 PknE3 PknE4 PknF1 PknF2 PknF3 PknF4 PknF5 PknG PknH PknJ PknL
c)
Mmar^rfp (RNAseq − Exp)
d)
Mmar^rfp (RNAseq − Stat)
pknA pknB pknD1 pknD2 pknE pknF1 pknF2 pknF3 pknF4 pknF5 pknG pknH1 pknH2 pknH3 pknH4 pknH5 pknH6 pknK1 pknK2 pknL pknI
e)
Mmuc^T (RNAseq − Exp)
f)
Mmuc^T (RNAseq − Stat)
g)
Mmuc^T (RNAseq − Exp)
h)
Mmuc^T (RNAseq − Stat)
0
10
20
30
i)
Mmuc^T (RNAseq)
log2(Stat/Exp)
−4
−2
0
2
4
mce1A mce1B mce1C mce1D mce1E mce1F mce3A mce3B mce3C mce3D mce3E mce3F mce3F_1 mce3E_1 mce3D_1 mce3C_1 mce3B_1 mce3A_1 mce4F mce4E mce4D mce4C mce4B mce4A mce5F mce5E mce5D mce5C mce5B mce5A mce7A mce7B mce7C mce7D mce7E mce7F mce8A mce8B mce8C mce8D mce8E mce8F

*Figure S7 Analysis of rifampin ADP-ribosyltransferase (*arr*) mRNA levels at different growth conditions*

(a) Change, expressed as log_2-fold change, comparing *arr* mRNA (MMUC_4701 and MMUC_1098) levels in exponentially growing and stationary *Mmuc*^{T} cells. A negative log_2-value suggests that the corresponding mRNA is more abundant in exponentially growing cells while a positive value suggest higher levels in stationary cells.

(b and c) Distribution profiles for *Mmuc*^{T} MMUC_4701 and MMUC_1098 mRNAs at different growth conditions expressed as percentage as indicated, panel (b) exponentially growing cells and panel (c) stationary cells. For details see main text.

Statistical significance, see Methods; *: $p < 0.05$; **: $p < 0.01$; ***: $p < 0.001$.

Fig S7

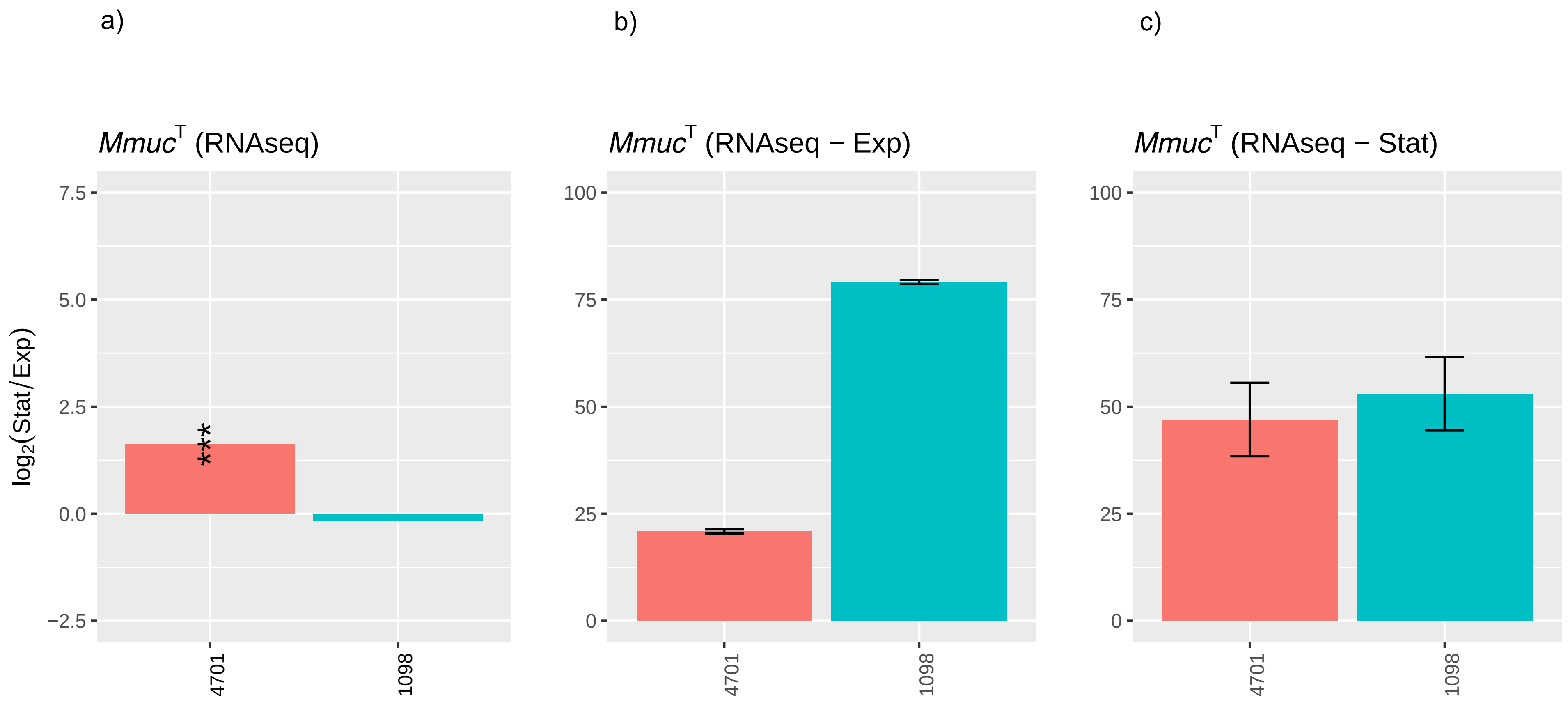

a)
b)
c)
Mmuc^T (RNAseq)
Mmuc^T (RNAseq − Exp)
Mmuc^T (RNAseq − Stat)
log2(Stat/Exp)
7.5
5.0
2.5
0.0
−2.5
***
100
75
50
25
0
4701
1098

Figure S8 Colony morphotypes analysis

Colony morphotypes, smooth (S) and rough (R), for *Mmuc*^{T}, *Mpho*^{T}, *Maub*^{T}, *Mneo*^{T} and *Mcos*^{T} as indicated. The different mycobacteria were grown on 7H10 media and incubated at respective optimal growth temperatures for 10 days: *Mmuc*^{T} (37°C), *Mpho*^{T} (30°C), *Maub*^{T} (30°C), *Mneo*^{T} (37°C) and *Mcos*^{T} (37°C).

Fig S8

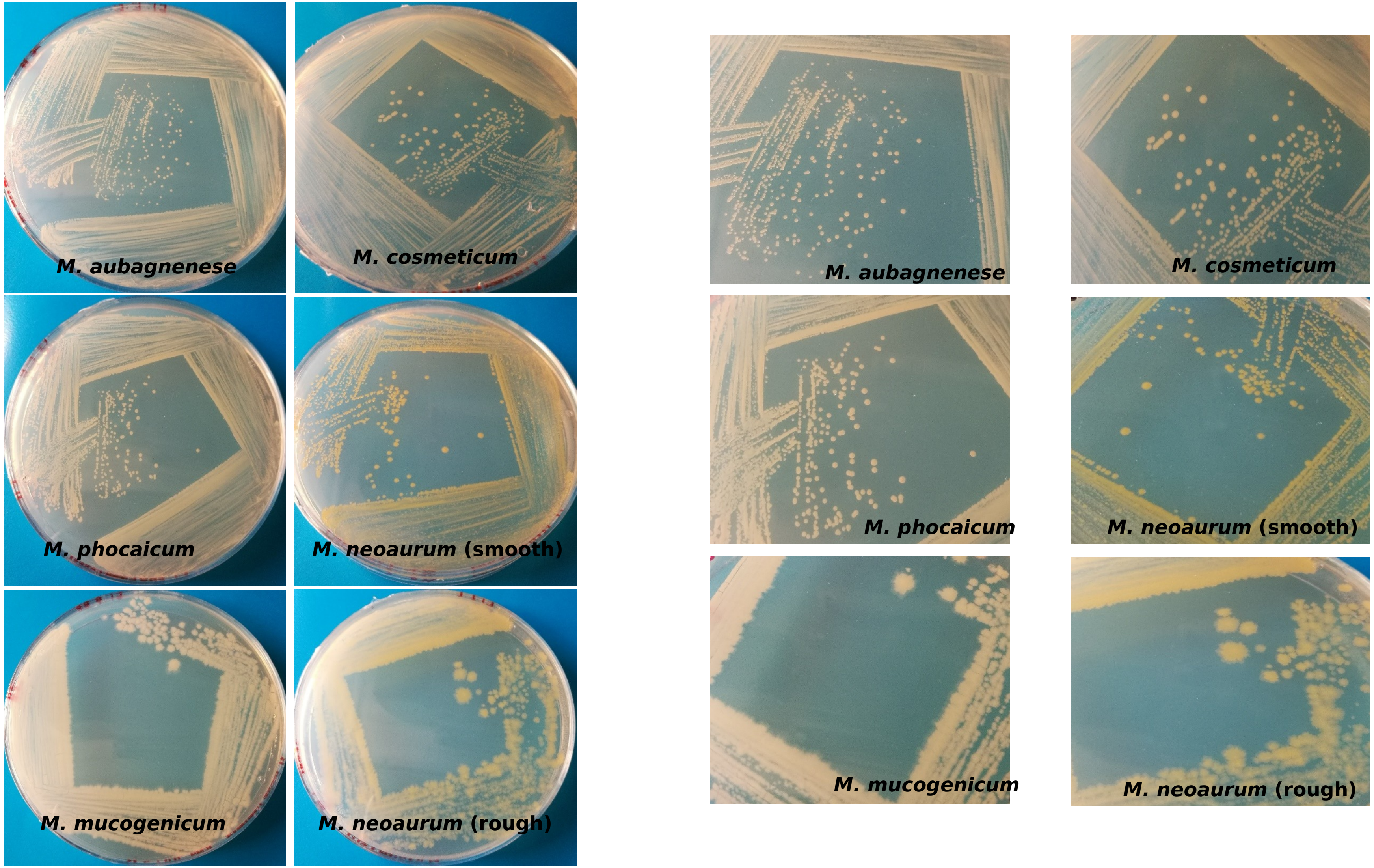

M. aubagnenese
M. cosmeticum
M. phocaicum
M. neoaurum (smooth)
M. mucogenicum
M. neoaurum (rough)
M. aubagnenese
M. cosmeticum
M. phocaicum
M. neoaurum (smooth)
M. mucogenicum
M. neoaurum (rough)
